# Emergence of Large-Scale Cell Morphology and Movement from Local Actin Filament Growth Dynamics

**DOI:** 10.1371/journal.pbio.0050233

**Published:** 2007-08-28

**Authors:** Catherine I Lacayo, Zachary Pincus, Martijn M VanDuijn, Cyrus A Wilson, Daniel A Fletcher, Frank B Gertler, Alex Mogilner, Julie A Theriot

**Affiliations:** 1 Department of Biochemistry, Stanford University, Stanford, California, United States of America; 2 Program in Biomedical Informatics, Stanford University, Stanford, California, United States of America; 3 Department of Bioengineering, University of California, Berkeley, California, United States of America; 4 Department of Biology, Massachusetts Institute of Technology, Cambridge, Massachusetts, United States of America; 5 Department of Neurobiology, Physiology and Behavior, University of California Davis, Davis, California, United States of America; 6 Department of Mathematics, University of California Davis, Davis, California, United States of America; 7 Department of Microbiology and Immunology, Stanford University, Stanford, California, United States of America; University of Washington, United States of America

## Abstract

Variations in cell migration and morphology are consequences of changes in underlying cytoskeletal organization and dynamics. We investigated how these large-scale cellular events emerge as direct consequences of small-scale cytoskeletal molecular activities. Because the properties of the actin cytoskeleton can be modulated by actin-remodeling proteins, we quantitatively examined how one such family of proteins, enabled/vasodilator-stimulated phosphoprotein (Ena/VASP), affects the migration and morphology of epithelial fish keratocytes. Keratocytes generally migrate persistently while exhibiting a characteristic smooth-edged “canoe” shape, but may also exhibit less regular morphologies and less persistent movement. When we observed that the smooth-edged canoe keratocyte morphology correlated with enrichment of Ena/VASP at the leading edge, we mislocalized and overexpressed Ena/VASP proteins and found that this led to changes in the morphology and movement persistence of cells within a population. Thus, local changes in actin filament dynamics due to Ena/VASP activity directly caused changes in cell morphology, which is coupled to the motile behavior of keratocytes. We also characterized the range of natural cell-to-cell variation within a population by using measurable morphological and behavioral features—cell shape, leading-edge shape, filamentous actin (F-actin) distribution, cell speed, and directional persistence—that we have found to correlate with each other to describe a spectrum of coordinated phenotypes based on Ena/VASP enrichment at the leading edge. This spectrum stretched from smooth-edged, canoe-shaped keratocytes—which had VASP highly enriched at their leading edges and migrated fast with straight trajectories—to more irregular, rounder cells migrating slower with less directional persistence and low levels of VASP at their leading edges. We developed a mathematical model that accounts for these coordinated cell-shape and behavior phenotypes as large-scale consequences of kinetic contributions of VASP to actin filament growth and protection from capping at the leading edge. This work shows that the local effects of actin-remodeling proteins on cytoskeletal dynamics and organization can manifest as global modifications of the shape and behavior of migrating cells and that mathematical modeling can elucidate these large-scale cell behaviors from knowledge of detailed multiscale protein interactions.

## Author Summary

The shape of animal cells is largely determined by the organization of their internal structural elements, including the filamentous structures of their cytoskeleton. Motile cells that crawl across solid substrates must assemble their cytoskeletal actin filaments in a spatially organized way, such that net filament growth and cell protrusion occur at the front of the cell. Actin filament dynamics, in turn, influence the overall shape of the cell by pushing on the plasma membrane. In this work, we have explored the ways that variations in small-scale actin filament growth dynamics are coupled to large-scale changes in cell shape and behavior. By manipulating the availability of a family of actin-binding proteins (Ena/VASP) that regulate actin filament growth, we can alter the overall cell shape and motile behavior of epithelial fish keratocytes—unusually fast-moving and regularly shaped cells. We have also found that unperturbed keratocytes in a population exhibit a continuum of shape and behavioral variations that can be correlated with differences in Ena/VASP levels. We have developed a mathematical model that allows us to explain our observations of intrinsic cell-to-cell shape variation, motile behavior, and cell responses to molecular perturbations as a function of actin filament growth dynamics in motile cells.

## Abstract 3

Mathematical modeling predicts global modifications of the shape and behavior of migrating cells from knowledge of detailed multiscale protein interactions.

## Introduction

The spatiotemporal coordination of the assembly, disassembly, and organization of the actin cytoskeleton is essential for efficient cell migration. The underlying mechanisms by which the actin cytoskeleton is organized and remodeled into specific architectures, which are then conveyed over large scales into observable cell morphologies, remain unclear. However, careful observation of large-scale morphology and behavior can shed light on these mechanisms. The heterogeneity of wild-type populations [1] can be used as a “natural experiment” in which potentially meaningful correlations between observations at molecular and global scales are determined. Because of the complex relationships between the underlying molecular interactions and observable parameters, physical and mathematical modeling is often necessary to interpret such quantitative data in terms of fundamental molecular mechanisms [2]. To achieve a mechanistic understanding of how the global shape and migratory behavior of cells are generated, we used a combination of quantitative analysis of natural cell-to-cell variation and mathematical modeling to help us grasp how the large-scale organization and function of the actin meshwork emerges and propagates from the dynamics of its molecular components.

The actin cytoskeleton can be remodeled by many different families of proteins, including the enabled/vasodilator-stimulated phosphoprotein (Ena/VASP) family, which affects dynamic processes such as growth, capping, and bundling of actin filaments [3], thereby regulating the local spatial organization of the actin cytoskeleton in cells [4–7]. Members of this family—represented by VASP, Mena (mammalian Ena), and EVL (Ena/VASP-like protein) in mammals—are largely functionally interchangeable [8] and have been recognized as important regulators of the actin cytoskeleton during cell migration and axon growth, as well as during filopodia formation, platelet aggregation, and phagocytosis [4,5,7,9–13].

Ena/VASP proteins have been of special interest in the field of cell migration, because they have been found to be both positive and negative regulators of cell speed in diverse motile cell types ranging from the actin-based movement of the intracellular pathogen Listeria monocytogenes to overall amoeboid migration of eukaryotic cells. The central proline-rich region of the *Listeria* surface protein ActA binds Ena/VASP proteins [14,15], which in turn recruit profilin–actin complexes [9,16] that are necessary for efficient actin monomer addition to growing filaments supporting bacterial propulsion. This mechanism accounts for the dramatic decrease in speed observed in *Listeria* when Ena/VASP proteins are depleted [8,17] and the speed increase observed when VASP is added to a reconstituted motility system [18]. Analogously, suppression of Ena/VASP protein function has been shown to decrease the speed of migrating neutrophils [10] and chemotaxis efficiency by Dictyostelium discoideum[11]. Conversely, Ena/VASP protein depletion resulted in faster moving fibroblasts due to the reorganization of the actin network, which became highly branched with short actin filaments, leading to more persistent lamellipodial protrusion [5,6]. A functional mechanism for these proteins has emerged, suggesting that Ena/VASP proteins remodel actin networks by enhancing the formation of long actin filaments, competing with capping protein, and potentially decreasing the branching activity of the actin-related protein 2/3 (Arp2/3) complex [4,6,7,19,20]. However, additional studies found no evidence for the latter two activities of VASP [21,22], and its exact molecular functions remain controversial.

Cell morphology represents the global manifestation of the cell's structural organization of the cytoskeleton and thus reflects the specific migratory behavior of different cell types. For example, epithelial fish keratocytes, which are among the fastest locomoting cells, exhibit flat lamellipodia as they glide along two-dimensional surfaces, whereas neutrophils have thicker, more amorphous pseudopodia that allow them to crawl through three-dimensional tissues with speeds comparable to that of keratocytes [23]. Keratocytes have been described as a “fan-” or “canoe-” shaped, exhibiting minor variations in shape and direction during migration [24–26]. With their simple stereotyped shape, keratocytes have been regarded as a good model system to study shape in migrating cells [26–29]. However, not all migrating keratocytes in culture are perfectly stereotyped; a certain fraction naturally exhibits more irregular morphologies [30–32] that have not been studied as well. Following our initial observation that these keratocyte morphologies were correlated with the presence or absence of VASP at the leading edge of the lamellipodium, we investigated how Ena/VASP activity influenced cell morphology as well as motile behavior. We hypothesized that the specific actin filament dynamics produced by actin remodeling proteins, such as Ena/VASP, organize the actin network and contribute to global cell morphology and migration. Quantitative analytical approaches were necessary to discern relationships between numerous perceptible morphological phenotypes and also to detect subtle changes caused by molecular manipulations.

To confirm our initial observation, we measured cell shape, leading-edge shape, filamentous actin (F-actin) distribution, cell speed, directional persistence, and VASP enrichment at the leading edge in a population of keratocytes. Systematic quantitative analysis revealed that these parameters correlated with VASP enrichment at the leading edge, spanning a clear continuum of coordinated phenotypes. Moreover, we have developed a mathematical model that explains the properties of this continuum—in particular, the quantitative correlations observed between the observable, large-scale parameters—in terms of small-scale molecular interactions between VASP and the growing actin architecture. Specifically, our model suggests that the role of Ena/VASP in protecting growing filaments allows for larger-scale cohesion in the actin meshwork, promoting smooth canoe shapes and faster migration. By experimentally manipulating Ena/VASP availability at the leading edge and thus local actin filament growth kinetics due to Ena/VASP activity, we were able to alter the prevailing morphology and trajectory of keratocytes within a population in a way that was accurately predicted by our model. Together, our results suggest that Ena/VASP proteins play a major role in cell morphology and motility by modulating the organization and thus promoting the large-scale coherence of the actin network. Our general approach of using detailed mathematical modeling to connect quantitative measurements of large-scale cell morphological and behavioral features to specific protein biochemical activities should be broadly applicable to many cytoskeleton-associated proteins involved in cell migration.

## Results

### VASP Is Enriched at “Smooth” and Not “Rough” Leading Edges of Epithelial Fish Keratocytes

**Figure 1:**
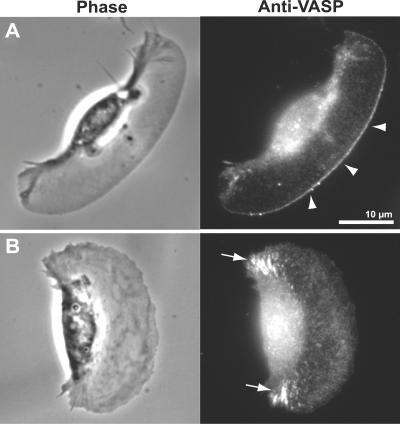
Strong VASP Localization at the Leading Edge Is Observed in Keratocytes with Smooth Leading-Edge Morphology A population of primary keratocytes is heterogeneous in morphology. (A) Keratocytes can have a smooth leading edge, showing strong VASP immunofluorescence that appears as a thin line at leading edge (arrowheads). (B) Keratocytes may also have rough leading edges with weak or absent VASP at the leading edge. VASP also appears localized at focal adhesions (arrows), which are more apparent in cells with rough leading edges. Immunofluorescence was performed using polyclonal anti-murine VASP antibodies. Scale bar = 10 μm.

Populations of primary migrating epithelial fish keratocytes are heterogeneous in cellular morphologies, sizes, and motile behaviors. Most descriptions of keratocytes focus on a subpopulation of cells with stereotyped canoe-like shapes [24–26] and smooth lamellipodial leading edges; however, many have more irregular shapes and rough leading edges [31,32] (Figure 1A and 1B). We initially examined cichlid keratocytes with these extreme morphologies and focused on their leading-edge morphology, which we classified by eye as smooth or rough.

Differences in morphology became more evident when we observed by immunofluorescence that VASP was localized as a uniform thin line at the leading edge of keratocytes with smooth leading edges and did not appear at the edge of cells with rough margins (Figure 1A and 1B). VASP was especially evident at focal adhesions at the rear sides of the cell body of rough polarized keratocytes (Figure 1B) and in keratocytes found in epithelial sheets (unpublished data). When we examined enhanced green fluorescent protein (EGFP)-VASP expression in live migrating keratocytes, we observed a similar localization, with VASP more strongly localized at smooth leading edges (Figure S1). Similar results were observed when the localization of EVL, a different member of the Ena/VASP family, was examined (unpublished data); however, we decided to focus on VASP because its function has been more thoroughly characterized. When individual migrating keratocytes expressing EGFP-VASP spontaneously switched from rough to smooth morphologies, an increase in VASP fluorescence at the leading edge and a decrease at focal adhesions was observed when keratocytes achieved the smooth morphology (Figure S1). Morphology switching was generally an uncommon event on the time scales over which time-lapse sequences were collected (tens of minutes), suggesting that the correlation between VASP localization and cell morphology is stable over the time scale of actin filament turnover in these cells (<30 s) [33]. Our observations, which suggested a relationship between Ena/VASP localization at the leading edge and large-scale cell morphology, prompted us to investigate whether VASP redistribution caused these morphological changes.

### Ena/VASP Availability at the Leading Edge Influences Cell Morphology and Motile Behavior

**Figure 2:**
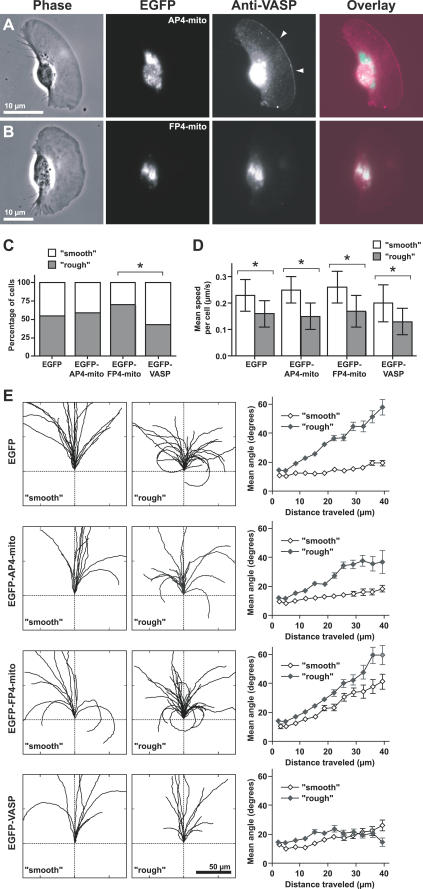
Ena/VASP Mislocalization Decreases the Prevalence of Fast, Persistent-Moving Smooth Cells (A) EGFP-AP4-mito (negative control) binds to mitochondria in the cell body, but does not mislocalize VASP, which, by immunofluorescence, appears as a thin line at the leading edge of cells with a smooth morphology (arrowheads). (B) EGFP-FP4-mito mislocalizes VASP at the surface of mitochondria thus preventing its function at the leading edge. Weak VASP localization was only observed at the leading edge in two cells out of more than 50 cells examined. Scale bar = 10 μm. (C) Keratocytes were classified as either having a smooth or rough leading edge within populations of migrating keratocytes expressing the aforementioned EGFP-tagged constructs. Cells with a rough leading-edge morphology are more prevalent than those classified as having a smooth morphology when EGFP-FP4-mito is expressed (70%) compared with controls, EGFP (55%) and EGFP-AP4-mito (59%). The incidence of rough keratocytes is significantly lower (43%) when EGFP-VASP is overexpressed (when compared to EGFP-FP4-mito, *p* < 0.05). (D) Keratocytes exhibiting smooth leading edges are significantly faster than those with rough leading-edge morphology (EGFP, *p* = 0.0002; EGFP-AP4-mito, *p =* 0.0004; EGFP-FP4-mito, *p* = 0.0004; EGFP-VASP, *p* = 0.0065). Mean and SD are plotted. (E) To perform qualitative comparisons of keratocyte turning during migration, trajectories of smooth or rough keratocytes were reoriented to start at *x,y =* 0 in the +*y* direction in standardized coordinate systems (left and middle panels). Trajectories were truncated (*x,y* limits = 150 μm) for illustration purposes. The distance between tick marks is 50 μm. For quantitative comparisons, mean angles between velocity vectors separated by specific distances traveled by cells were plotted. Larger mean angles correspond to increased curvature in the trajectories of migrating cells (right panels). Mean angles are significantly smaller for smooth keratocytes expressing negative control constructs compared to rough cells (EGFP, *p* = 0.0003; EGFP-AP4-mito, *p =* 0.001), showing that smooth keratocytes maintained straighter paths than rough ones. Ena/VASP protein mislocalization using EGFP-FP4-mito causes smooth cells to move in more curved trajectories with larger mean angles not significantly different from those of rough cells and significantly different from those of smooth cells expressing control constructs (compared to EGFP smooth, *p =* 0.01; EGFP-AP4-mito smooth, *p* = 0.005). On the other hand, when keratocytes overexpress EGFP-VASP, rough keratocytes, which have increased trajectory curvature in controls, have straighter paths with smaller angles similar to those from smooth cells expressing controls and EGFP-VASP. Mean and SEM are plotted.

To test whether Ena/VASP proteins directly modulated leading-edge shape, we manipulated their availability at the leading edge of keratocytes. To decrease Ena/VASP availability, we used a construct (FP4-mito) derived from the *Listeria* ActA protein, which localizes to mitochondria when expressed in eukaryotic cells [15,34] and has four proline-rich repeats (P4) that efficiently bind Ena/VASP proteins [14,15,35]. FP4-mito was previously shown to function as an Ena/VASP dominant-negative construct by sequestering and mislocalizing Ena/VASP proteins at the surface of mitochondria thus preventing their function at their normal sites of activity, such as the leading edge, tips of filopodia, and cell–cell contacts in tissue culture cells as well as in developing embryos [4,5,36–38]. As a control, we used a similar construct (AP4-mito) that has been previously used as negative control [4,5,36–38] because it contains point mutations that dramatically reduce binding to Ena/VASP proteins [39] while retaining the ability to localize to mitochondria [5]. When we expressed EGFP tagged FP4-mito in keratocytes, VASP (Figure 2A and 2B) and EVL (unpublished data) were efficiently mislocalized to mitochondria, and a higher percentage of migrating keratocytes, which were observed with time-lapse video-microscopy, exhibited the rough morphology (70%) compared with controls (Figure 2C). Conversely, when EGFP-VASP was overexpressed, a significantly lower percentage of keratocytes (43%) exhibited the rough phenotype (compared to cells expressing EGFP-FP4-mito, *p* = 0.03, Figure 2C). These results suggest that VASP enrichment at the leading edge can tilt the balance of morphology toward the smooth phenotype.

We also used these time-lapse sequences to examine differences in motile behavior between cells with smooth and rough morphologies. When we measured migration speed, we found that smooth cells were significantly faster than rough cells (Figure 2D, *p* < 0.01) suggesting that lamellipodial morphology, which can be influenced by VASP availability at the leading edge, is tightly coupled to the migration speed of these cells. Because fish keratocytes have been observed to generally migrate with persistent straight trajectories over long distances in vitro [40], we examined whether directional persistence was related to morphology. We found that smooth cells expressing control constructs (EGFP and EGFP-AP4-mito) had significantly straighter trajectories compared with those of rough cells (*p* < 0.001, Figure 2E).

Since Ena/VASP availability influenced the fraction of smooth, straight-moving keratocytes within a population, we next examined whether manipulating VASP availability at the leading edge would alter cell trajectories. When Ena/VASP proteins were mislocalized (EGFP-FP4-mito), smooth cells moved in more curved trajectories that were similar to those of rough cells and significantly different from smooth cells expressing control constructs (*p* < 0.001, Figure 2E). In contrast, when EGFP-VASP was overexpressed, rough keratocytes, which had curved trajectories in controls, maintained straighter trajectories similar to those from smooth cells expressing control constructs and EGFP-VASP. These results suggest that directional persistence was more sensitive to VASP availability at the leading edge than was leading-edge morphology. Taken as a whole, our results show that VASP localization at the leading edge correlates with smooth, fast, and straight-moving keratocytes, and that manipulating Ena/VASP availability alters the morphology and trajectory curvature of keratocytes within a population.

### Keratocytes with Canoe Shapes, Smooth Leading Edges, and High VASP at the Leading Edge Make Up One Extreme of a Continuum of Phenotypic Morphologies

Thus far, we had observed that VASP localization was related to broad classes of keratocyte leading-edge morphologies and that we could manipulate morphology by mislocalizing or overexpressing VASP. We wondered whether morphological variation among wild-type keratocytes might be related to VASP levels at the leading edge, and therefore we performed a detailed, quantitative characterization of a keratocyte population. Instead of using a binary and subjective classification of smooth versus rough, we characterized the natural morphological heterogeneity of keratocytes along several measurable and objective phenotypic continua.

**Figure 3:**
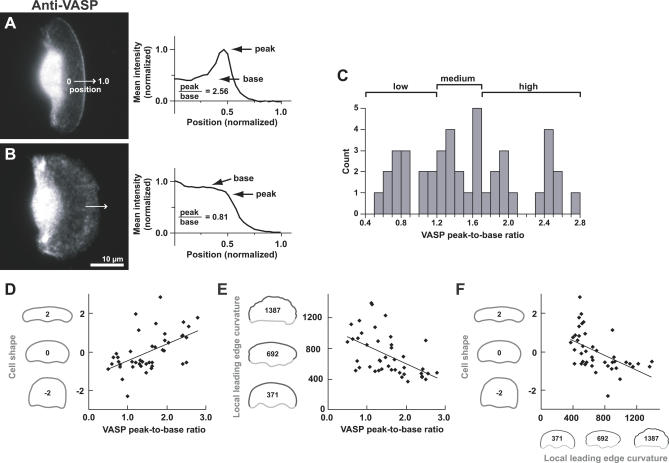
Keratocytes with High VASP at the Leading Edge Are Canoe-Shaped with Smooth Leading Edges (A, B) Using immunofluorescence, VASP intensity levels were measured along lines (∼5 μm wide) positioned across lamellipodia in the middle of cells roughly perpendicular to the leading edge of keratocytes (arrows). The relative levels of VASP at the leading edge of a population of keratocytes were compared using VASP peak-to-base ratios, which were calculated by dividing the highest mean fluorescence intensity at the leading edge (peak) by the lowest mean intensity found interior to the leading edge (base). For cells with low VASP at the leading edge, peak and base positions were assigned based on mean positions from cells (*n* = 30) with medium or high VASP peak-to-base ratios and thus clear peak and base positions. (A) An intensity linescan shows that VASP is enriched at the leading edge of a smooth cell with a VASP peak-to-base ratio of 2.56. (B) On the other hand, the rough leading edge of a keratocyte has a VASP peak-to-base ratio of 0.81 and thus VASP is practically absent from the leading edge. Scale bar=10 μm. (C) The keratocyte population examined (*n =* 43) had a wide range of VASP peak-to-base ratio values ranging from 0.5 to 2.8. (D) The shapes of keratocytes were compared using PCA, which identified the major modes of shape variation of polygonal cell contours extracted from intensity-thresholded fluorescent images. A shape mode value of zero corresponds to the mean shape and the negative or positive values correspond to standard deviations describing canoe (+SD) or round D shape (−SD) on the *y-*axis. Representative cell contours for the specified values are shown on the *y*-axis. For example, the cells in (A) and (B) have shape mode values of 1.32 and −0.77, showing that they can be quantitatively described as canoe or D shaped, respectively. This shape mode significantly correlates with VASP peak-to-base ratios (*r^2^* = 0.28, *p* = 0.0002, *n =* 43, solid line). (E) To compare leading-edge shapes, we measured and normalized their degree of local curvature. Local leading-edge curvature negatively correlates (*r^2^* = 0.28, *p* = 0.0003, *n =* 43, solid line) with VASP peak-to-base ratios. (F) A significant negative correlation is also observed between cell shape and local leading-edge curvature (*r^2^* = 0.28, *p* = 0.0003, *n =* 43, solid line).

To measure cell morphology rigorously, we determined mathematically the major modes of shape variation by applying the principal components analysis (PCA) to a population of keratocyte shapes represented as aligned, polygonal contours [41]. We found that three primary modes of shape variability accounted for over 95% of all morphological variation: one mode corresponding approximately to cell size, one corresponding to aspect ratio (i.e., whether cells were shaped more like a wide canoe or a rounded “D”), and one corresponding to the position of the cell body along the front–rear direction (see Materials and Methods, Figure S2) [41]. Since we wanted to test whether cell morphology was related to VASP levels, we quantified VASP enrichment at the leading edge of cells by dividing the highest mean VASP fluorescence intensity across the leading edge (“peak”) by the lowest VASP mean intensity (“base”) found interior to the leading edge. This measure of “VASP peak-to-base ratio” is illustrated in Figure 3A and 3B. We found that the population of keratocytes examined (*n* = 43) displayed a wide and apparently continuous range of VASP peak-to-base ratios (Figure 3C). When we compared cell morphology to VASP enrichment at the leading edge, we found that only the shape mode that correlated with VASP levels described the canoe-to-rounder-D–shape transition. Keratocytes with VASP enriched at the leading edge (high VASP peak-to-base ratios) had a tendency to resemble canoe shapes, whereas cells with low VASP at the edge were more likely to have rounder D shapes (*p* = 0.0002, *n =* 43, Figure 3D).

To evaluate the shapes of leading edges quantitatively instead of qualitatively classifying them as smooth or rough, we measured the degree of roughness of the leading edges by calculating the sum of the local curvature at each of 90 points along front of the cell contours (see Materials and Methods). Since curvature at a point is defined as the reciprocal of the radius of the osculating circle, sharply bending curves that are present in rough leading edges osculate small circles and thus have large local curvatures. Our results confirmed our qualitative observation (from Figure 1) that strong VASP localization at the leading edge correlated with smooth edges (*p* = 0.0003, *n* = 43, Figure 3E). Additionally, canoe-shaped keratocytes had decreased local curvature and thus smooth leading edges (*p* = 0.0003, *n =* 43, Figure 3F). In summary, enrichment of VASP at the leading edge correlated with canoe shape and smooth leading edges, strongly suggesting a morphological continuum related to VASP activity at the lamellipodial edge.

**Figure 4:**
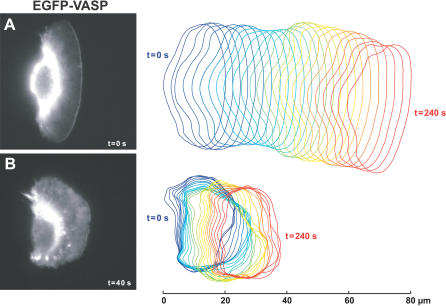
Smooth, Canoe-Shaped Keratocytes Migrate “Coherently,” with Fast Speeds and Persistent Shapes Outlines of migrating keratocytes overexpressing EGFP-VASP were generated from each frame of time-lapse image sequences separated by 10 s to examine the shape of cells as they migrate. Outlines are colored from blue to red to represent time (0–240 s), superimposed, and plotted on the same scale for visual comparison. Speed can be estimated from the distances traveled by each keratocyte because outlines correspond to the same total time. Fluorescent images correspond to the first (A) and fifth (B) frames of each time-lapse sequence and are scaled to match to the outlines. (A) The leading edge and overall shape of a smooth, coherent keratocyte does not vary extensively as the cell migrates. (B) In the case of a rough and rounder keratocyte, outlines show that the leading edge changes shape rapidly and widely. This keratocyte is unable to maintain persistent coordinated protrusion of its lamellipodium. Specific segments of the leading edge extend forward while adjacent regions lag behind (notice blue and orange outlines). This keratocyte migrates at approximately half the speed of the coherent keratocyte in (A)*.*

To examine the behavior of live keratocytes with smooth or rough leading edges, we followed their contours, which were generated from each frame of time-lapse sequences of keratocytes overexpressing EGFP-VASP. The shape of the leading edge in rough cells varied widely, whereas smooth cells maintained a constant shape with minor fluctuations (Figure 4 Video S1). In the particular example shown (Figure 4), we also observed that the smooth keratocyte migrated at approximately twice the speed of the rough one.

Our results indicated that the five parameters considered thus far—VASP peak-to-base ratio, cell shape, local leading edge curvature, speed, and directional persistence—all correlated with each other, creating a continuum of keratocyte phenotypic morphologies. One extreme of this continuum contained fast, straight-moving cells with VASP enriched at the leading edge, canoe-like shapes, and smooth leading edges (Figure 2E 2F, and S3). We refer to cells in this end of the continuum as “coherent” to convey their stable morphology and directed movement. The opposite extreme in the continuum of keratocyte morphologies encompassed slow, meandering cells with low VASP at the leading edge, rounder D shape, and rough leading edges, which we denote as “decoherent.”

### The Distributions of VASP and F-actin, Which Correlate along the Leading Edge, Are Peaked for Coherent and Flat for Decoherent Keratocytes

**Figure 5:**
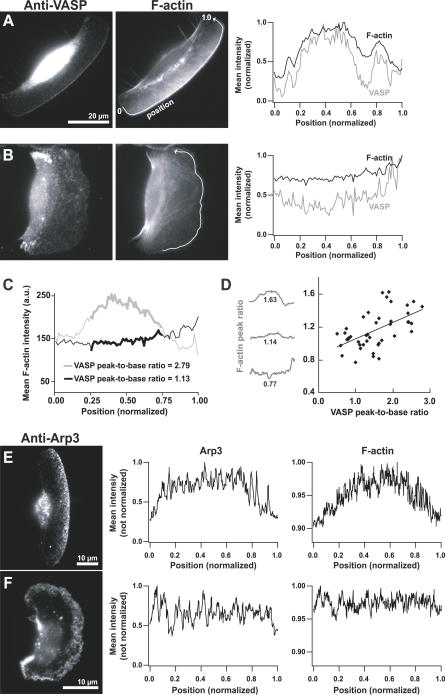
VASP Enrichment at the Leading Edge Correlates with Peaked F-Actin Distributions (A) The distributions of VASP (gray line in the graph) and F-actin (black line in the graph) were measured along the length of the leading edge of cells (position indicated by the arrow). The cell shown has VASP enriched at its smooth leading edge (VASP peak-to-base ratio = 1.84). The distribution of F-actin along the leading edge of this smooth cell is peaked in the middle and strongly correlates with that of VASP (*r^2^* = 0.82, *p* < 0.0001). Scale bar = 20 μm. (B) A keratocyte with rough leading edge and very weak VASP at the leading edge (VASP peak-to-base ratio = 0.76) has a very flat distribution of VASP (gray line in the graph) and F-actin (solid black line in the graph) measured along the leading edge (arrow). The distributions of VASP and F-actin along the leading edge of this cell strongly correlate with each other (*r^2^* = 0.81, *p* < 0.0001). Additionally, the distributions of VASP and F-actin of the entire population of keratocytes strongly correlate (<*r>* = 0.71, SD = 0.23, *p* < 0.0001, *n* = 43). (C) The F-actin density along the leading edge of a representative coherent cell with high VASP peak-to-base ratio—2.79 (gray line)—is increased compared to that of a decoherent cell with low VASP peak-to-base ratio—1.13 (black line). Mean intensity values are not normalized and were obtained from keratocytes imaged from the same coverslip. F-actin distributions between different cells were compared by calculating a ratio (F-actin peak ratio) of the mean F-actin intensity values from the middle half of the leading edge (0.25 to 0.75 position along the edge, indicated by the thick regions of each line in the graph), which generally correspond to the highest intensity values in peaked F-actin distributions, to the mean of the F-actin values from the rest of the leading edge (positions 0 to 0.25 and 0.75 to 1.00 along the edge, indicated by the thin regions of each line). Cells with peaked F-actin distributions had larger F-actin peak ratios than did cells with flat distributions. The F-actin peak ratio of the coherent cell (gray line) is 1.45, whereas that of the decoherent cell (black line) is 0.94. Also compare the cells in (A) and** (**B), which have F-actin peak ratios of 1.56 and 0.94, respectively. (D) VASP peak-to-base ratios significantly correlate with F-actin peak ratios (*r^2^* = 0.31, *p* = 0.0001, *n =* 43, solid line). (E, F) For comparisons of F-actin and Arp3, mean intensity values were measured along lines (∼0.5 μm wide) positioned along the leading edge of keratocytes, immediately interior to cell edge. Anti-Arp3 mean fluorescence intensities measured along the leading edge of keratocytes are consistent with those of F-actin. A representative smooth cell (E) exhibits peaked Arp3 and F-actin distributions along the leading edge while a rough cell (F) has flat distributions. Scale bar = 10 μm.

Because previous studies have indicated that keratocyte leading-edge shape may be related to actin filament (F-actin) density [28], we compared the distribution of F-actin to keratocyte morphology and VASP levels at the leading edge. Keratocytes with high VASP and a coherent morphology had F-actin distributions along the leading edge that peaked in the middle at the front of the cell (Figure 5A), whereas cells with low VASP and a decoherent morphology had uniform F-actin distributions (Figure 5B). We also found that the F-actin density along the leading edge of coherent cells was increased compared with decoherent cells (Figure 5C). To compare VASP enrichment to the enhancement of F-actin in the middle of the leading edge of different cells, we calculated a ratio (referred to as “F-actin peak ratio”) of the mean F-actin intensity values from the middle half of the leading edge (1/4 to 3/4 position along the edge) to the mean of the F-actin values from the rest of the leading edge (positions 0 to 1/4 and 3/4 to 1 along the edge) of each cell (Figure 5C). We found a significant correlation between F-actin enhancement in the middle of the leading edge (F-actin peak ratios) and VASP enrichment (VASP peak-to-base ratios), suggesting that VASP accumulation at the leading edge is associated with the peaked or graded accumulation of F-actin in coherent cells (*p* < 0.0001, *n* = 43, Figure 5D).

When we examined the relationship of the Arp2/3 complex to F-actin and cell morphology, we found that Arp3 distribution, as measured by immunofluorescence, corresponded to that of F-actin in both coherent and decoherent cells, which had peaked and flat distributions, respectively (Figure 5E and 5F). When we compared the spatial distribution of the ratio of Arp3 to F-actin to infer the degree of filament branching, no consistent differences in Arp3–to–F-actin ratios were observed between different keratocyte morphologies (unpublished data), suggesting that VASP activity does not significantly affect branching in keratocyte lamellipodia, consistent with previous findings using purified protein systems [21,22], but in contrast to other studies employing cells or purified proteins [4,6,19].

### Mathematical Modeling Reveals How VASP Activity—F-Actin Growth Enhancement by Protection from Capping—Determines the Coherent Phenotype

To unify our observations into a functional context, we developed a mathematical model that accounted for self-organization of keratocyte leading edge and VASP-mediated F-actin growth dynamics. This model allowed us to make predictions about keratocyte shape and was based on the following assumptions about actin dynamics and protrusion at the leading edge:

(1) The F-actin network is organized in a dendritic array such that actin filaments are oriented at ±35° relative to the locally normal direction of protrusion [42]. Filaments are distributed over a wide range of angles, but this distribution is doubly-enhanced and peaked at ±35° due to optimal growth conditions for both mother and daughter filaments, the angle between which is 70°. Since mother and daughter filaments are oriented at the same angle with respect to the leading edge [42], we lump all filaments growing to the left and to the right into two groups, and do not explicitly keep track of individual angles.

(2) Growing barbed ends at the leading edge elongate with a rate limited by membrane resistance and local concentration of actin monomers (G-actin) [43].

(3) Arp2/3-mediated filament branching takes place with equal rate per each existent leading-edge filament [28] (Text S1). This per filament rate is equal to the total number of filaments nucleated over the whole leading edge per second divided by the total number of the uncapped leading-edge filaments. The molecular pathway determining this rate is unknown; a plausible mechanism could be based on rapidly diffusive molecules, the total number of which is conserved, controlling the total number of branching events per cell. Assuming that the branching takes place only along the leading edge, each filament has equal probability to become a mother filament. Then, as the total number of growing filament ends increases, the branching rate per filament inversely decreases. A filament at +35° branches off filaments oriented at −35°, and vice versa [42]. We define the leading-edge filament as the filament whose growing barbed end is in physical contact with the membrane.

(4) VASP associates with/dissociates from barbed ends with constant rates and remains associated with elongating barbed ends until it dissociates [6,20,44].

(5) VASP protects barbed ends from capping; unprotected barbed ends are capped at a constant rate [6,20].

(6) The barbed ends of elongating actin filaments undergo lateral flow along the leading edge with a rate proportional to local protrusion [28,45].

(7) The shape of the leading edge is determined by the graded radial extension model [29], according to which the local slope of the leading edge is determined by the ratio of the local normal protrusion rate to that in middle front of the cell.

(8) The length of the leading edge is a constant parameter. At the sides of the leading edge, boundary densities of the uncapped (VASP-free and VASP-associated) barbed ends are constant parameters in the model. These parameters are crucial for the model predictions (discussed below).

**Figure 6:**
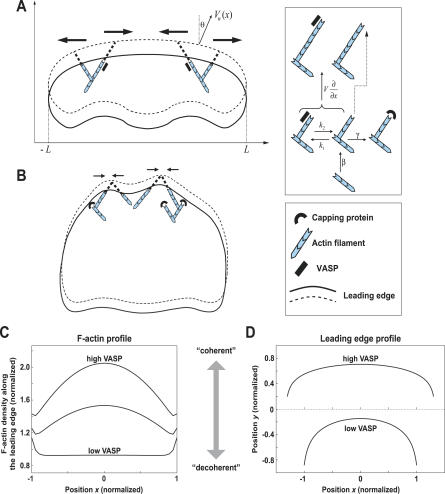
Mathematical Modeling Explains the Coherent and Decoherent Keratocyte Phenotypes (A) In coherent cells, long actin filaments are protected from capping and undergo significant lateral flow (arrows), smoothing heterogeneities at the leading edge. According to the Graded Radial Extension model, *V*_n_(*x*)=*V* cosθ, where *V* represents cell speed, *V*_n_ represents the local protrusion rate, and θ is the orientation of the normal to the leading edge at position *x*. Quantitative actin dynamics (right) are explained in the Materials and Methods and Text S1. (B) In decoherent cells, short filaments that are not protected from capping undergo less-extensive lateral flow (arrows) and may focus into heterogeneities at the leading edge causing the unstable protrusion of microregions. (C) Barbed end density and nascent filament branching were chosen so that when VASP activity is low, the F-actin density along the leading edge appears flat (bottom curve). When high VASP activity was entered into our model, the F-actin density along the leading edge emerged as an inverted parabola (top curve), with F-actin density peaked in the middle, as observed experimentally in coherent cells. Position is normalized by the half-length of the leading edge. The prediction that peaked F-actin density is proportional to the level of VASP is qualitatively consistent with the experiment (see Figures 4C and 4D). (D) Based on protrusion rate as a function of barbed end distribution along the leading edge, the computed leading edge profile is wide (canoe-shaped) in coherent cells with high VASP at the leading edge (top curve) and short (D shaped) in decoherent cells with low VASP at the leading edge (bottom curve). Position (*x*) is normalized by the half-length of the leading edge of the short, decoherent cell. The same scale is used for the coherent cell, which is 30% longer, so the corresponding profile extends beyond 1.

These assumptions, which are expressed mathematically in Text S1, lead to equations governing VASP activity, F-actin density, protrusion rate, and leading-edge stability and shape. The analytical and numerical solutions qualitatively explain our experimental observations as follows. In coherent cells, which have high VASP activity at the leading edge and low effective capping rate, the average density of actin filament barbed ends at the leading edge is increased, as well as the proportional VASP density associated with these ends (see Figure 5A–5D; F-actin density, measured along the curve very close to the leading edge in this figure, is proportional to the number of actin filaments per micrometer intersecting with the curve parallel and just behind the leading edge, and therefore is also proportional to the density of barbed ends, assuming that all filaments abutting the leading edge are growing [42]). A simple explanation for this increase in F-actin density in the presence of VASP is that VASP skews the balance between branching and capping. Without VASP, nascent barbed ends emerge at a constant rate, whereas a constant capping rate maintains an average number of growing filaments. VASP protects a fraction of the growing barbed ends from capping, so the effective capping rate per total number of growing ends decreases, increasing the average number of growing filaments (see Text S1 for quantitative details). In addition, when VASP is enriched at the leading edge, actin filaments, which grow for longer time periods before capping, undergo significant lateral flow (illustrated in Figure 6A).

When we investigated the stability of the leading edge of coherent keratocytes mathematically, we found that high VASP activity maintains greater density of barbed ends abutting the membrane at the front, leading to low membrane resistance per filament. This low resistance allows the protrusion rate to become insensitive to F-actin density, and instead limited by G-actin concentration. The even distribution of G-actin along the leading edge, together with the lateral flow of actin filaments, leads to the smooth leading edge of coherent cells (Text S1). In this coherent regime, significant fluctuations of the F-actin density do not cause respective fluctuations of the local protrusion rate, and the leading edge remains smooth. In decoherent cells, which have low VASP activity at the leading edge and a high effective capping rate, elongating filaments are rapidly removed from the leading edge by capping and the density of barbed ends decreases (Figure 5C). Barbed ends, which grow for shorter time periods before capping, undergo slow lateral flow and are not redistributed along the leading edge. In this decoherent regime, fluctuations of F-actin density cause respective fluctuations of the local protrusion rate: high local branching density due to stochastic fluctuations at random locations increases the number of filaments pushing the membrane at the front creating a local protrusive “lobe” (Figure 6B and Text S1). Barbed ends slide faster into and slower out of the lobe, creating a positive feedback between actin filament local focusing and protrusion that causes short-scale instabilities of the leading edge, thus making its shape rough.

When we modeled the F-actin profiles along the leading edge of cells, we found that they depended crucially on the boundary conditions at the sides of the leading edge and on the total branching rate. We assumed that at the sides of the leading edge the cell, where the large adhesions are located, there are specific local conditions generating and maintaining a constant density of uncapped barbed ends. If this fixed boundary density is equal to the average density being maintained along the leading edge by the dynamic balance between branching and capping, then the F-actin density along the leading edge is constant (Text S1). However, if the boundary density is less than this threshold, more nascent filaments branch out closer to the center of the cell. This, in turn, increases the net branching rate at the center, because more nascent filaments branch off the higher number of the existent filaments at the center. The existent growing barbed ends start to effectively compete for resources (because the total number of branching events per second is conserved), and if the F-actin density at the cell sides is kept lower, the center “wins.” This positive feedback leads to the characteristic inverted parabolic profile of the F-actin distribution along the leading edge with maximum at the center (Figure 6C) that matches our observations (see Figure 5A and 5C). The lateral flow is crucial for maintaining the coherent inverted parabolic profile of the F-actin distribution along the leading edge; without it, the barbed ends would cluster irregularly at random locations. The flat F-actin distribution at the leading edge of decoherent cells is due, in part, to the slow and irregular lateral flow along the leading edge.

The characteristic canoe shape of coherent cells is achieved through a graded distribution of extension along the leading edge. Experimentally, we observed that coherent cells with high VASP at the leading edge have increased F-actin density at the leading edge (Figure 5C), which according to our model, leads to increased rates of actin growth and protrusion (Figure E of Text S1). With this high F-actin density peaking in the middle of the leading edge, the rate of protrusion, which is insensitive to the density of barbed ends, decreases very slowly along the leading edge, so the leading edge remains flat and extends far from side to side creating the characteristic wide canoe shape (Figure 6D). At the sides, where the F-actin density decreases significantly, membrane resistance starts to limit protrusion, and the rapidly decreasing protrusion rate leads to high curvature at the sides of the leading edge. In decoherent cells, the overall shape of the leading edge remains parabolic, although with sharper transitions from the center to the curved sides, which are apparent as a rounder D keratocyte leading edge shape (Figure 6D). Because these cells are characterized by lower F-actin densities, which correspond to a qualitatively different region in the density–velocity relation compared with coherent cells (Figure E of Text S1), the protrusion rate in decoherent cells decreases faster from the center to the sides, where protrusion drops to levels that cannot overcome membrane resistance. Consequently, the distance from the center to the sides is less than that in coherent cells, so decoherent cells are narrower from side to side.

### VASP Localization at the Leading Edge by Association with Barbed Ends Is Necessary To Achieve the Coherent Phenotype in Keratocytes

**Figure 7:**
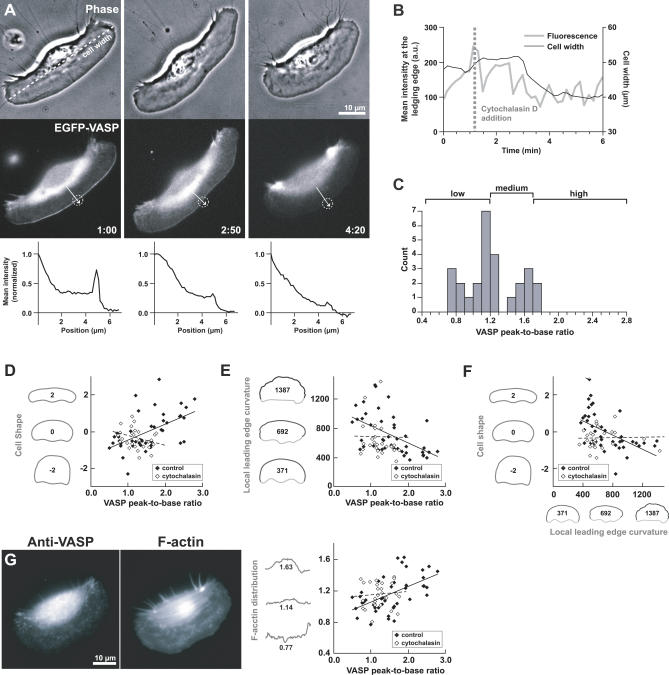
Cytochalasin Delocalizes VASP from the Leading Edge, Changing the F-Actin Network and Cell Shape (A) Time-lapse images show that overexpressed EGFP-VASP, which is enriched at the leading edge before addition of 1.0 μM cytochalasin D (time: 1:00, left panel), becomes weaker at the leading edge ∼2 min after cytochalasin treatment (time: 2:50, middle panel). Cytochalasin D was added at 70 s (∼1.2 min). Approximately 3 min after cytochalasin treatment (time: 4:20, right panel), EGFP-VASP can barely be seen at the leading edge of this keratocyte that begins to exhibit a D shape rather than the original canoe shape. Time = min:s. Scale bar = 10 μm. Fluorescence intensities measured across the leading edge (arrows) quantitatively confirm the enrichment of EGFP-VASP at the leading edge before cytochalasin treatment and its delocalization a few minutes after addition of the drug (bottom graphs). (B) The levels of EGFP-VASP at the leading edge and the width of the keratocyte in (A) were compared as a function of time. Cell width (axis perpendicular to migration) was measured from one side of the cell to the other (dotted line in (A)). EGFP-VASP intensity levels were measured inside a circle (4 μm diameter) placed in the middle of the cell at the leading edge (dotted circles in (A)) in each frame of the time-lapse sequence. These two parameters temporally correlate with each other (*r^2^ =* 0.21, *p* = 0.0009). The levels of EGFP-VASP at the leading edge (thick gray line) dramatically drop ∼2 min after cytochalasin addition (time ∼3 min, dotted line), followed by a decrease in cell width (thin black line). (C) The frequencies of VASP peak-to-base ratios obtained from immunofluorescence images of keratocytes treated with cytochalasin D confirm that VASP becomes displaced from the leading edge. The keratocyte population with high VASP peak-to-base ratios observed in wild-type cells (see Figure 3C) becomes absent after cytochalasin treatment, leaving only cells with low and medium values. (D) Shape mode analysis reveals that cytochalasin treatment (open diamonds) eliminates the population of keratocytes with canoe shapes (>1 in *y-*axis) compared to control cells (closed diamonds) causing most cells to resemble the mean shape of the population or rounder D shapes. No significant correlation was found between this shape mode and enrichment of VASP at the leading edge (VASP peak-to-base ratio) in cytochalasin treated cells (*r^2^* = 0.09, *n =* 27, dashed line) compared to control cells. (E) Cytochalasin treatment (open diamonds) eliminates the correlation between local leading-edge curvature and VASP enrichment at the leading edge (*r^2^* = 0.0004, *n =* 27, dashed line) previously observed in control cells (closed diamonds). (F) The negative correlation between cell shape and local leading edge curvature observed in control cells (closed diamonds) is abolished in cells treated with cytochalasin (open diamonds, *r^2^* = 0.0009, *n =* 27, dashed line). (G) After cytochalasin treatment, a typical cell has VASP absent from the leading edge (VASP peak-to-base ratio = 0.83) and an F-actin peak ratio of 0.96 corresponding to a flat F-actin distribution along the leading edge. Cytochalasin treatment eliminated cells with peaked F-actin distributions corresponding to high F-actin peak values from our population. In addition, no significant correlation was found between F-actin peak ratios and enrichment of VASP at the leading edge (VASP peak-to-base ratio) in our population of cytochalasin treated cells (*r^2^* = 0.006, *n =* 27, dashed line) compared to control cells. Scale bar = 10 μm.

Since our model predicted that VASP was responsible for the morphological phenotypes observed, we tested our model by acutely delocalizing Ena/VASP proteins from the leading edge of keratocytes. VASP was delocalized by competition with the pharmacological barbed end capper, cytochalasin D [6,46]. VASP delocalization was often accompanied by a decrease in cell width, suggesting that these two parameters were functionally connected (Figure 7A, B). This result also supported our model, which proposed that low VASP activity at the leading edge resulted in narrow D shaped keratocytes (see Figure 6C, D). In a population of keratocytes, cytochalasin treatment eliminated cells with highest enrichment of VASP at the leading edge (Figure 7C).

Our quantitative comparison of shape showed that cytochalasin treatment eliminated keratocytes with extreme canoe shapes (Figure 7D). Moreover, the observed correlations that established a relationship between cell shape, local leading edge curvature, F-actin distribution, and VASP enrichment at the leading edge of wild-type cells were absent in cells treated with cytochalasin (Figure 7D–7G). Our results show that cytochalasin D, acting as a barbed end capper, antagonized VASP localization at the leading edge and altered the shape of keratocytes and the F-actin network towards the decoherent side of the phenotypic continuum.

### The Edges of Nascent Lamellipodia Are Rough and Accumulate VASP in Protruding Microregions That Mature into Smooth Lamellipodia

**Figure 8:**
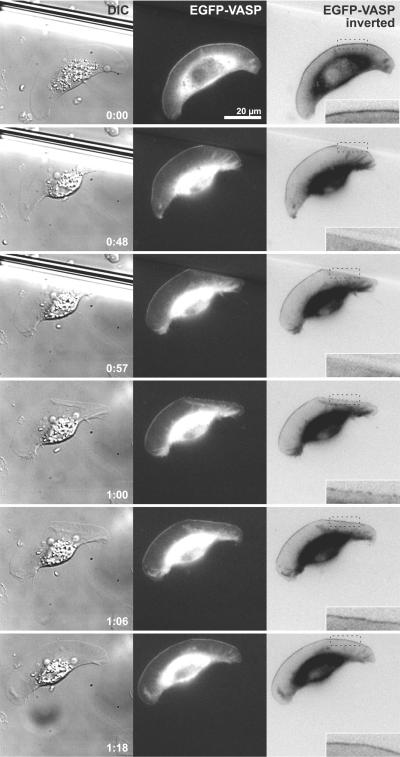
Nascent Lamellipodia Emerge with Rough Edges Accumulating VASP in Microprotrusions Eventually Maturing into a Smooth Morphology We developed a method to generate nascent lamellipodia during recovery of protrusion after temporarily stalling a section of the lamellipodium. A glass micropipette, acting as a barrier, was lowered into the path of movement of a migrating keratocyte overexpressing EGFP-VASP and forced down until a section flexed parallel to the surface. The pipette was left in place until the cell was in firm contact with the pipette and subsequently removed by translating the pipette in the direction of cell migration. A coherent keratocyte shows EGFP-VASP as a uniform thin line at the leading (time: 0:00, min:s). Fluorescent images are shown in both regular and inverted contrast for clarity. Insets show inversed and zoomed in images of the corresponding boxed areas of the leading edge. When this keratocyte reaches the edge of the barrier, the lamellipodium temporarily stops protruding forward and acquires a very flat shape corresponding to the shape of the barrier (time: 0:48, 0:57). The cell continues migrating in the original direction of motion while EGFP-VASP becomes displaced from the edge of the region in contact with the barrier (see inset). When the micropipette barrier is removed (between 0:57 and 1:00), the leading edge of the lamellipodium immediately resumes protrusion and appears rough with several protruding microregions enriched in EGFP-VASP (time: 1:00). The levels of EGFP-VASP quickly recover along the impacted region and become uniform (time: 1:06). Only 18 s after removal of the barrier, the keratocyte's original shape and EGFP-VASP localization at the leading edge are restored (time: 1:18). Scale bar = 20 μm.

During extensive observation of different keratocyte morphologies, we hypothesized that coherent keratocytes with high VASP at the leading edge represented a mature state of cellular organization and migration. We evaluated the contribution of VASP in the generation of smooth lamellipodia in coherent cells by obstructing lamellipodial protrusion and examining its subsequent emergence and recovery. When we placed a barrier in the path of movement of a coherent keratocyte with EGFP-VASP enriched at the leading edge, the front edge of the lamellipodium that reached the barrier became temporarily stalled and the levels of VASP at the leading edge dramatically decreased (Figure 8 and Video S2). When the barrier was removed, the leading edge instantly resumed protrusion and appeared rough with protruding microregions enriched in EGFP-VASP. EGFP-VASP quickly became uniform as the cell continued to regain the original smooth leading edge shape. This rapid redistribution of VASP and thus barbed ends along the leading edge confirms the previously described phenomenon of lateral flow, which is important for the maintenance of coherence, as suggested by our model (Figure 6).

## Discussion

### Ena/VASP Remodeling of the Actin Cytoskeleton Leads to Morphological Differences in Keratocytes

Ena/VASP proteins have not only been implicated in the global determination of migration speeds in different cell types [5,6,10,11,17], but also affect the spatial organization of local actin-related cellular structures, such as lamellipodia that contain a branched dendritic network or filopodia, which possess long actin filaments. The ultrastructure of wild-type lamellipodia and growth cones has revealed long actin filaments, whereas those with depleted Ena/VASP revealed shorter, more branched filaments [4,6]. Lamellipodial structure may also be reorganized to give rise to filopodia by altering actin filament length, through changes in the activities of cappers and antagonizing factors that facilitate filament growth [7,44,47]. Therefore, the balance between the activity of Ena/VASP proteins and capping proteins may determine the type of actin network architecture present in different cell types, which may be observed as changes in cell morphology.

Our initial observations of epithelial fish keratocytes revolved around cell shape and leading-edge morphology. Keratocytes have broad, flat lamellipodia that lack filopodia and have been generally described as having a characteristic fan or canoe shape [24–26] despite the fact that morphological variation is part of the natural heterogeneity of keratocytes obtained from primary cultures [31,32]. Particularly, very little attention has been devoted to less-regular morphologies and to understanding how “coherent,” smooth keratocytes differ from “decoherent,” rough ones. In this study, we have shown molecular differences between these two extreme morphologies and established a strongly correlated suite of morphological phenotypes related to Ena/VASP accumulation at the leading edge. Coherent keratocytes have VASP that is enriched at the leading edge and peaked F-actin distributions along the edge, whereas decoherent cells have sparse VASP and flat F-actin distributions, suggesting that VASP activity at the leading edge modulates the architecture of the actin network, which then becomes evident as the morphological and motile phenotypes observed.

EGFP-VASP delocalization from the leading edge of keratocytes after cytochalasin D treatment showed that Ena/VASP proteins might be binding at or near the barbed end of actin filaments, in agreement with a previous study in fibroblasts, which proposed that this mechanism protects actin filament barbed ends from capping [6]. This proposed anticapping activity of Ena/VASP has been controversial: biochemical studies have demonstrated that Ena/VASP proteins can inhibit actin filament capping by several different barbed binding proteins [20], whereas other in vitro studies showed no evidence of such competition by VASP [21,22,48]. Even though the net result of Ena/VASP activity appears to result in increased actin filament length, the in vivo molecular mechanism of this effect is still unclear. Increased actin filament length by Ena/VASP proteins may stem from direct competition with capping protein for barbed end binding, increased actin filament growth rate, reduced filament branch formation, or a combination of any of these activities [6,19–21]. Our results are more consistent with the hypothesis that a primary function of VASP at the leading edge is to oppose the activity of capping proteins.

A mathematical model helped us understand how the underlying actin network organization and dynamics were influenced by these VASP activities and how that could lead to distinct cellular morphologies**.** This model pointed to a specific molecular mechanism by which VASP activity increases the length of filaments within the actin network: VASP prevents filaments from being capped, thus allowing them to grow for a longer time. We also experimentally tested the prediction that VASP was needed for the maintenance of the coherent phenotype based on the mechanistic assumption that VASP competes with capping. We treated cells with cytochalasin D to antagonize barbed-end binding by VASP and thus increase filament capping. We observed a drop in VASP density at the leading edge after cytochalasin D treatment and, in agreement with our model, the side-to-side lamellipodial width decreased linearly with a rate of ∼0.1 μm/s, similar to that of the inward actin flow (C.A.W., P.T. Yam, L. Ji, K. Keren, G. Danuser, and J.A.T., unpublished data)]. The decrease in VASP levels at the edge continued for a few tens of seconds during which the keratocyte width shrank by 20%–30%, and then stabilized. Moreover, VASP displacement from the leading edge not only decreased cell width, but also eliminated cells with extreme coherent canoe-shaped morphologies. Together with the altered fraction of keratocyte morphologies observed after VASP mislocalization or overexpression, these data support the idea that VASP activity is important for the maintenance of the coherent morphology.

### Morphology Is Coupled to the Motile Behavior of Keratocytes

When keratocyte migration was examined as a function of cell morphology, we found that coherent, smooth cells migrated significantly faster than decoherent, rough cells, which demonstrates that cell morphology is tightly coupled to the speed of migrating keratocytes. These results are consistent with previous descriptions of keratocytes with fast protrusion rates as fan-shaped, whereas cells with slower protrusion rates were described as irregular or fibroblast-like in shape [30]. Our mathematical model suggests that the increased F-actin density at the leading edge, which is observed in coherent cells, creates less resistance per filament as the filament elongates, so the rates of F-actin growth and protrusion accelerate (Figure E of Text S1), leading to the observed faster migration speed. In decoherent cells, which have low F-actin density resulting from low VASP activity at the leading edge, the membrane resistance per filament is large and becomes the limiting factor in the protrusion rate, which becomes very sensitive to the F-actin density and thus cells migrate slower. We observed a strong relationship between keratocyte speed and morphology, which depended on VASP localization at the leading edge. A positive correlation between VASP localization and cell speed or protrusion has also been observed in *Dictyostelium* [11] and melanoma cells [49], contrary to observations in fibroblasts [5,6]. These conflicting observations suggest that different cell types may distinctly coordinate protrusion with overall cell migration and may have different rate limiting parameters of actin dynamics and cell motility.

When we examined the directional component of velocity in keratocytes, we observed that rough, decoherent, wild-type keratocytes had increased curvature of trajectory compared to smooth, coherent, wild-type keratocytes. Unlike the smooth and regular leading edge of coherent keratocytes, the leading edge of decoherent cells can fluctuate widely during protrusion. In other words, different regions of the leading edge may protrude at different rates in an uncoordinated fashion. This phenomenon may be associated with greater frequency of cell turning, because either the whole left or right half of the lamellipodium would advance faster than the other half, effectively changing the average orientation of the leading edge and consequently changing the direction of migration. Thus, morphological variations manifest themselves during cell migration creating different behavioral patterns. We also found that Ena/VASP protein mislocalization led to increased trajectory curvature. This result is consistent with previous studies showing that intracellular *Listeria* that were deficient in Ena/VASP recruitment exhibited increased trajectory curvature [50] and VASP-null *Dictyostelium* displayed decreased cell directionality during chemotaxis [11]. Note, however, that directional control may be mechanistically quite different in these cell types.

### Concluding Remarks

Epithelial fish keratocytes can rapidly migrate in a graceful gliding motion, all the while maintaining a relatively uniform and persistent shape. This migratory behavior requires the exquisite coordination of the intricate cellular migration machinery composed of three processes—protrusion, adhesion, and retraction—which are typically dissected separately. This work, in which we focused on the lamellipodial protrusive actin-based machinery resulting in the elongation and capping of actin filaments, is no exception. Future work, armed with broader and more detailed mathematical models, should strive to integrate our increasing understanding of these individual parts of the machinery and to understand how they interact to generate spatiotemporally coordinated cell migration in different cell types. We believe that this work, though limited in scope and susceptible to hidden variables and as-yet unknown molecular players, provides an example of how information from multiple spatial and organizational scales can be successfully brought together to explain part of a complex phenomenon.

Within the reductionist context of this work, quantitative analysis and mathematical modeling were crucial to the understanding of cell shape and motile behavior in terms of the molecular activity of Ena/VASP proteins. In view of the strong correlation between VASP enrichment at the leading edge and the quantitative morphological parameters analyzed in fixed cells, a more quantitative characterization of the morphology (shape, leading-edge curvature) of live migrating cells may be warranted in the future to provide more detailed insights about the dynamics and activity of Ena/VASP. It is important to note that even though our mathematical model was able to recapitulate and provide a self-consistent explanation of our quantitative observations of cell morphology, F-actin organization, and motile behavior, it was only able to do so in a qualitative manner. Ideally, future modeling will be able quantitatively bridge experimental data and theory. Some steps in this direction are discussed in Text S1. Nevertheless, our current model served as an important tool to generate a testable prediction and to interpret the cell morphologies observed.

Overall, cell morphology represents a large-scale manifestation of underlying cytoskeletal organization and dynamics. Regulation and modulation of the actin cytoskeleton are likely to be major biological mechanisms affecting cell migration. Actin-remodeling proteins localize to propulsive structures in morphologically diverse cell types—neutrophils, fibroblasts, neurons, and intracellular bacterial pathogens—where they play crucial roles in the morphogenesis and maintenance of pseudopods, lamellipodia, filopodia, or bacterial comet tails, all of which inherently have different actin network organizations. Ena/VASP proteins, which are capable of enhancing the elongation of actin filaments by competing with capping protein for barbed-end binding, have emerged as important actin-remodeling proteins and strong candidates for the modulation of the underlying actin cytoskeleton that dictates cell morphology and migration.

## Materials and Methods

### Keratocyte culture and transfection.

Keratocytes were cultured from the scales of the Central American cichlid Hypsophrys nicaraguensis as described [51], but the isolated scales were sandwiched between two acid-washed glass 25-mm coverslips and cultured at room temperature in the dark using Leibovitz's L-15 medium (Gibco BRL; http://www.invitrogen.com) supplemented with 14.2 mM HEPES pH 7.4, 10% FBS, and 1% antibiotic-antimycotic (Gibco BRL) before transfection or processing for immunofluorescence the day after isolation.

Keratocytes were transfected using a small-volume electroporator for adherent cells as previously described [52,53]. Coverslips containing keratocytes were placed in fish Hank's Balanced Salt Solution (HBSS) [54] containing 85% NaCl, and 20 μl of plasmid DNA (1 μg/μl) in water were placed directly onto the cells. Keratocytes were immediately electroporated with three pulses of 185 V and allowed to recover for ∼24 h in culture media to allow for expression. Before live cell imaging or immunofluorescence, sheets of keratocytes that had migrated off the scales were washed for ∼5 min in 85% PBS, 2.5 mM EGTA, pH 7.4 to separate individual migrating cells.

### Immunofluorescence.

Indirect immunofluorescence was performed using rabbit polyclonal anti-murine VASP (2010) and anti-murine EVL (1404) antibodies [4,5]. Keratocytes were fixed in ice cold 2.5% glutaraldehyde, 0.025% Triton X-100 in PBS for 1 min. Autofluorescence was quenched by incubation in 0.1% sodium borohydride in PBS twice for 5 min. Cells were blocked and permeabilized using PBS-BT (3% BSA, 0.1% Triton, 0.02% sodium azide in PBS) before incubation with antibodies diluted in PBS-BT. F-actin was labeled by incubation with fluorescently labeled phalloidin (Invitrogen; http://www.invitrogen.com).

Indirect Arp2/3 immunofluorescence was performed using rabbit polyclonal anti-human Arp3 antibodies as described previously [55,56], except that cells were simultaneously fixed and permeabilized in cytoskeleton buffer containing 0.32 M sucrose (CBS) [57], 4% formaldehyde, 0.1% Triton X-100, and 0.5 μg/ml FITC-phalloidin (Invitrogen) for 15 min.

Images were acquired using an Axioplan microscope (Carl Zeiss Microimaging; http://www.zeiss.com) equipped with a CCD camera (MicroMAX 512BFT; Princeton Instruments; http://www.piacton.com).

### Cloning.

FP4-mito, AP4-mito, and mouse VASP in pMSCV [5,17] were subcloned into pEGFP-C1 (Clontech Laboratories; http://www.clontech.com) using standard molecular biology techniques. BglII and HindIII restriction sites were used to subclone FP4-mito, AP4-mito, and murine EVL. HindIII and BspEI were used to subclone VASP.

### Pharmacological treatment.

Because individual keratocytes are heterogeneous in their responses to pharmacological agents, they were treated with 0.5 μM for 5 min; 0.8 μM for 2, 3, and 5 min; or 1.0 μM cytochalasin D (Sigma; http://www.sigmaaldrich.com) for 2 min in culture media.

### Time-lapse video microscopy and image analysis.

Time-lapse images were collected at 10-s intervals using a Nikon Diaphot-300 inverted microscope with a CCD camera (MicroMAX 512BFT; Princeton Instruments; http://www.piacton.com). The rear of keratocytes was tracked using the “Track Points” option of MetaMorph software (Molecular Devices; http://www.moleculardevices.com) to measure speed and direction as previously described [31,50,58]. For population speed analysis, tracks were truncated to correspond to the same time (150 s). In this study, a minority of cells imaged using a different objective (*n =* 11) and persistent circlers (*n =* 4) were excluded from trajectory analysis. The population used for trajectory analysis included: EGFP smooth, *n =* 20; EGFP rough, *n =* 23; AP4-mito smooth, *n =* 9; AP4-mito rough, *n =* 16; FP4-mito smooth, *n =* 11; FP4-mito rough, *n =* 26; VASP smooth, *n =* 9; and VASP rough, *n =* 11. Keratocyte leading-edge morphology was classified as smooth or rough by eye by determining whether each cell was more similar to the smooth or rough reference cells depicted in Figure 1A and 1B. Long trajectories were collected using a low-magnification air objective, which had a resolution suboptimal for detailed cell shape measurements.

To compare immunolocalized Arp3 and F-actin along the leading edge and the enrichment of immunolocalized VASP (VASP peak-to-base ratios) across the leading edge of keratocytes, measurements were obtained using the “linescan” option in MetaMorph and background subtracted. F-actin and VASP distributions along the leading edge were calculated using the cell outline polygons as guides (see “cell shape analysis” section below). For each vertex point along the leading edge of a given cell, intensities were sampled at 20 points (∼2 μm) for F-actin and ten points for VASP (∼1 μm ranging from ∼0.3 μm outside to ∼0.7 μm inside the outlines) spaced one pixel apart along the inward normal and averaged.

### Micropipette barrier experiments.

Micropipettes were pulled using a P-92 Flaming-Brown micropipette puller (Sutter Instruments; http://www.sutter.com) from 0.5 mm inner diameter (ID)/1.0mm outer diameter (OD) glass capillaries, and positioned with a Narishige MMO-202ND micromanipulator. Keratocytes were transfected overnight using FuGENE6 (Roche Diagnostics; http://www.roche.com) and allowed to recover and express EGFP-VASP for one day. Time-lapse images were acquired using a Zeiss Axiovert 200 inverted microscope with Nomarski differential interference contrast optics and a Cascade II 512B CCD camera (Photometrics; http://www.photomet.com).

### Cell shape analysis.

Cell morphology was measured by representing cell shapes as polygonal outlines and comparing those outlines with the PCA, as described [41]. Briefly, cell shapes were manually determined by using the “magnetic lasso” tool in Adobe Photoshop to trace the edge of each cell, based on images of fluorescent phalloidin. Each lasso selection was converted into a binary mask and outlines were extracted from those masks to derive a series of *(x,y)* points corresponding to the cell boundary. Each series was resampled to 150 points, evenly spaced along the cell boundary. Finally, the outlines were mutually aligned to bring the shapes into a common reference frame. The remaining variability in the point positions was then characterized with PCA to derive a small number of highly explanatory modes of shape variation.

This analysis determined that three principal “shape modes,” which are illustrated in Figure S2, are sufficient to explain over 95% of the variability in shape of the 43 untreated cells and 27 cytochalasin D–treated cells. Of these modes, only the second—describing shapes ranging from canoe to D—correlated with VASP distribution. To quantify the shape of an individual cell, we measured its position along this mode in terms of standard deviations from the mean shape.

To calculate the roughness of each cell's leading edge, we used a measure that we refer to as “local leading-edge curvature.” Mathematically, the curvature of a function at a particular point is defined as the reciprocal of the radius of the circle that has the same tangent as the function at that point. A sharply bending curve will share a tangent with a small circle, and thus have a large curvature; in the limit, a straight line is tangent to an infinitely large circle and has zero curvature. The curvature of a parametric plane curve [*x*(p),*y*(p)] at a point p can be calculated as (*x*′·*y* ′′ – *y*′·*x*′′)/(*x*′^2^+*y*′^2^)^3/2^, where prime signifies the first derivative at point p and double prime the second derivative. We calculated the curvature at each of 90 points along the leading edge of the keratocyte outlines, using central-difference approximations to the derivatives. To determine the values of “local leading-edge curvature,” we summed the absolute values of the curvatures along the leading edge, and multiplied this by the length of the leading edge to account for the fact that smaller keratocytes will have higher total curvature due to their size alone. (Under this measure, a perfectly smooth semicircle sampled at 90 points would have a value of 90π [≈283]). Overall, rough leading edges have high local leading-edge curvature values and smooth leading edges have low values.

To examine the contours of migrating keratocytes (Figure 4), cell outlines were calculated as described in P.T. Yam, et al. (unpublished data).

### Statistical comparisons.

The mean speeds per cell for each pair of transfected keratocyte populations (e.g., EGFP versus AP4-mito, EGFP versus FP4-mito, etc.) and for rough and smooth cells (e.g., EGFP rough versus EGFP smooth) were statistically compared using the Mann-Whitney test. Trajectories were evaluated by comparing mean angles between 2 and 45 μm (distance traveled) using the same test. The proportions of smooth and rough cells present in all combinations of populations of transfected keratocytes were compared using the two-sample test for binomial proportions [59]. Linear regression was used to compare the relationship between VASP peak-to-base ratios, cell shape, F-actin distribution (F-actin ratio), and local leading-edge curvature.

### Mathematical modeling.

Briefly, we modeled the densities of right- (left-) oriented growing barbed ends along the leading edge with functions b^+^(*x*,*t*) (b^−^(*x*,*t*)) for ends not associated with VASP and with functions b̃^+^(*x*, *t*) (b̃^−^(*x*, *t*)) for ends associated with VASP.

According to the model assumptions, the following equations govern these densities:

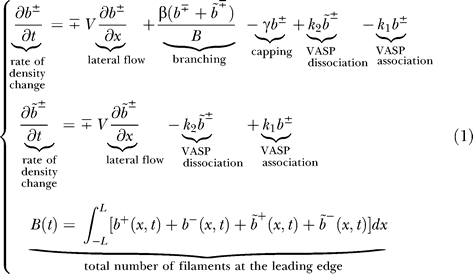



We considered these equations on the leading edge: −*L* ≤ *x* ≤ *L*. We choose the boundary conditions at *x* = ±*L* as follows:

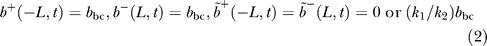



The meaning of these conditions, choice of the model parameters, and methods of solution of equations are thoroughly explained in Text S1.

We described the leading-edge profile with the function *y = f*(*x*). The overall steady shape is derived from the Graded Radial Extension model [28,29] according to the formula:

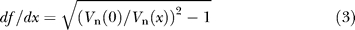
where

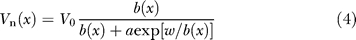
is the local protrusion rate, which is a function of the local density of barbed ends.

To investigate the local stability of the leading edge, we solved the system:

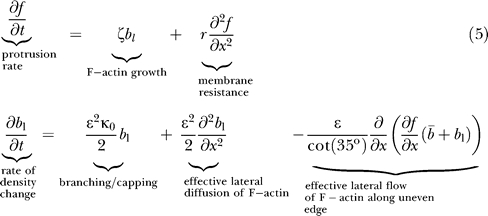
where *b̄* is the average density of barbed ends and *b*_l_ is the local density of barbed ends.

## Supporting Information

Figure S1 Keratocytes Can Switch Morphologies with VASP Appearing Strongly Localized as a Thin Line at the Leading Edge of Cells Exhibiting the Smooth Morphology A keratocyte overexpressing EGFP-VASP transitions from the rough to the smooth leading-edge morphology as it migrates is shown. EGFP-VASP appears more prominent in focal adhesions (arrowheads) throughout the lamellipodium when the cell exhibits a rough leading edge (time: 0:00, 0:50). Sparse amounts of EGFP-VASP can be observed at dynamic protrusive leading-edge microregions (time: 0:00, 0:50) that eventually coalesce and persist in time and space to achieve the smooth morphology (time: 8:13). Time = min:s. Scale bar = 10 μm.
(9.7 MB PDF)

Figure S2 PCA Reveals Modes of Keratocyte Shape Variation Cell morphology was measured by representing cell shapes as polygonal outlines and comparing those outlines with the principal components analysis. Three primary modes of shape variability accounted for over 95% of all morphological variation of the 43 untreated and 27 cytochalasin-treated cells: one mode corresponding to approximately to cell size (left), one corresponding to aspect ratio (i.e., whether cells were shaped more like a wide canoe or a rounded D, middle), and one corresponding to the position of the cell body along the front to back direction of the cell (right). Of these modes, only the second mode—describing shapes ranging from canoe to D— correlated with VASP distribution. To quantify the shape of an individual cell, we measured its position along each mode in terms of standard deviations (σ) from the mean shape (μ).
(169 KB PDF)

Figure S3 Four-Dimensional Graph of the Parameters Examined Shows That Keratocyte Lie within a Continuum of Morphological Phenotypes One extreme of the continuum contains cells with VASP enriched at the leading edge, canoe-shape, and smooth leading edges. High VASP peak-to-base ratios correspond to VASP enriched at the leading edge, high local leading-edge curvature values correspond to rough leading edges, and larger positive cell-shape values correspond to canoe shapes. The small dots on each plane are projections of the larger dots in the middle.
153 KB PDF).

Text S1 Details of Mathematical Model (2.6 MB PDF)

Video S1 Smooth, Canoe-Shaped Keratocytes Migrate “Coherently,” with Fast Speeds and Persistent Shapes, whereas Rough, D-Shaped Keratocytes Migrate “Decoherently,” with Slower Speeds and Fluctuating Shapes Smooth, coherent keratocytes (top), which have EGFP-VASP enriched as a thin line at the leading edge, maintain their shape and migrate faster than rough, decoherent cells (bottom). The coherent keratocyte shown migrates at approximately twice the speed of the decoherent one*.* The leading edge and overall shape of the decoherent cell changes rapidly and widely compared to the coherent one. Scale bar = 20 μm.
(8.6 MB MOV)

Video S2 Nascent Lamellipodia Emerge with Rough Edges and Rapidly Acquire a Smooth Morphology A glass micropipette, acting as a barrier, was lowered into the path of movement of a migrating, coherent keratocyte overexpressing EGFP-VASP. When the keratocyte reaches the barrier, the lamellipodium temporarily stops protruding forward, while EGFP-VASP becomes displaced from the edge of the region in contact with the barrier. When the micropipette barrier is removed (between 0:57 and 1:00), the leading edge of the lamellipodium immediately resumes protrusion and appears rough with several protruding microregions enriched in EGFP-VASP. Only 18 s after removal of the barrier, the keratocyte's original shape and uniform EGFP-VASP localization at the leading edge are restored. Time = min:s. Scale bar = 25 μm.
(9.5 MB MOV)

## Abbreviations

Arp: actin-related protein
EGFP: enhanced green fluorescent protein
Ena: enabled
EVL: Ena/VASP-like protein
F-actin: filamentous actin
PCA: principal component analysis
VASP: vasodilator-stimulated phosphoprotein
