# Supplementary material for: Emergence of Large-Scale Cell Morphology and Movement from Local Actin Filament Growth Dynamics
Source: PLoS Biol. 2007 Aug 28;5(9):e233. doi: 10.1371/journal.pbio.0050233 (PMC1951782; doi:10.1371/journal.pbio.0050233)
Supplement: Supplementary file 1 [file pbio.0050233.sg001.pdf]

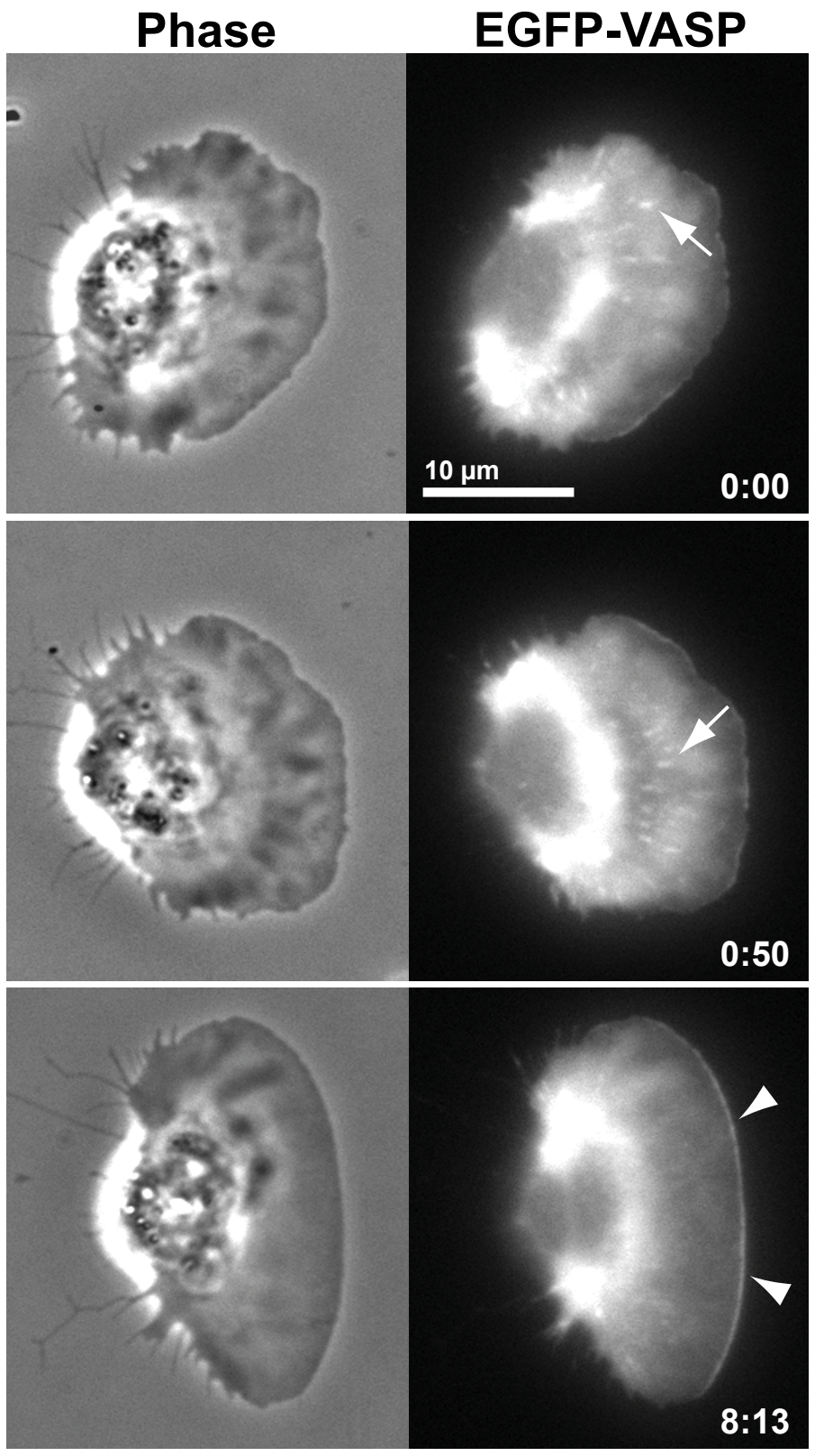

Phase
EGFP-VASP
10 μm
0:00
0:50
8:13
