# Supplementary material for: Emergence of Large-Scale Cell Morphology and Movement from Local Actin Filament Growth Dynamics
Source: PLoS Biol. 2007 Aug 28;5(9):e233. doi: 10.1371/journal.pbio.0050233 (PMC1951782; doi:10.1371/journal.pbio.0050233)
Supplement: Supplementary file 2 [file pbio.0050233.sg002.pdf]

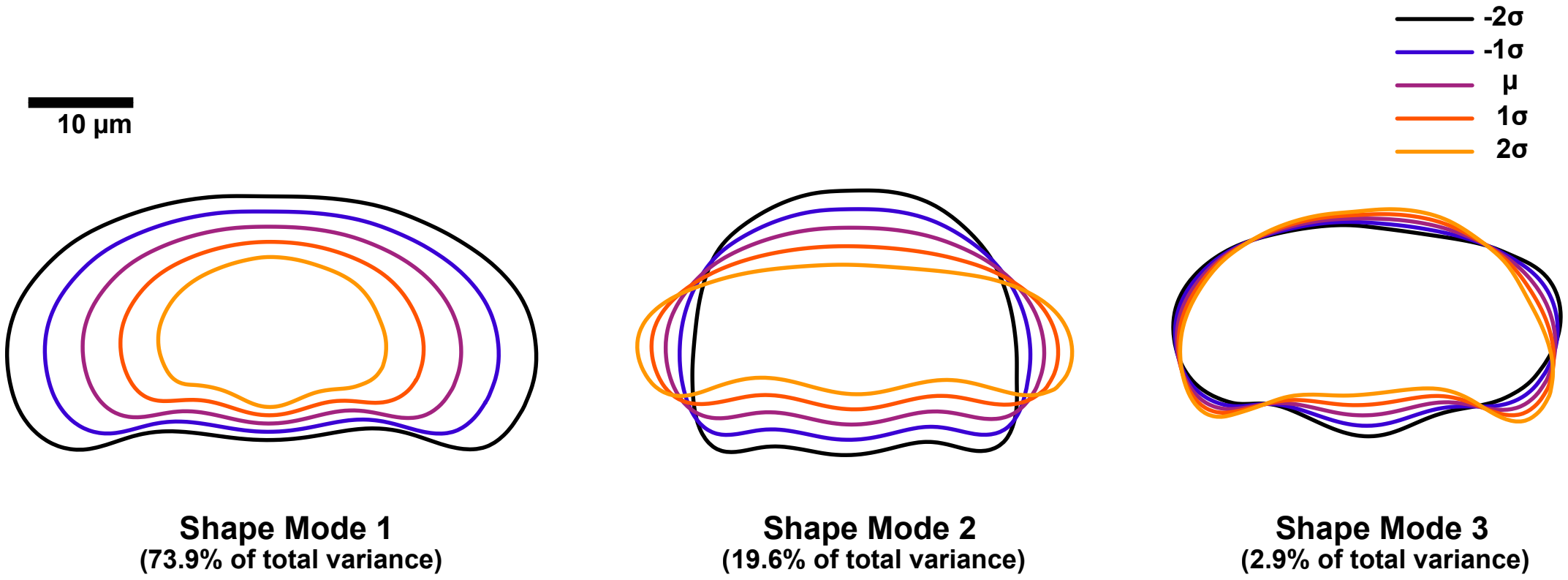

10 μm
-2σ
-1σ
μ
1σ
2σ
Shape Mode 1
(73.9% of total variance)
Shape Mode 2
(19.6% of total variance)
Shape Mode 3
(2.9% of total variance)
