# Supplementary material for: Emergence of Large-Scale Cell Morphology and Movement from Local Actin Filament Growth Dynamics
Source: PLoS Biol. 2007 Aug 28;5(9):e233. doi: 10.1371/journal.pbio.0050233 (PMC1951782; doi:10.1371/journal.pbio.0050233)
Supplement: Supplementary file 3 [file pbio.0050233.sg003.pdf]

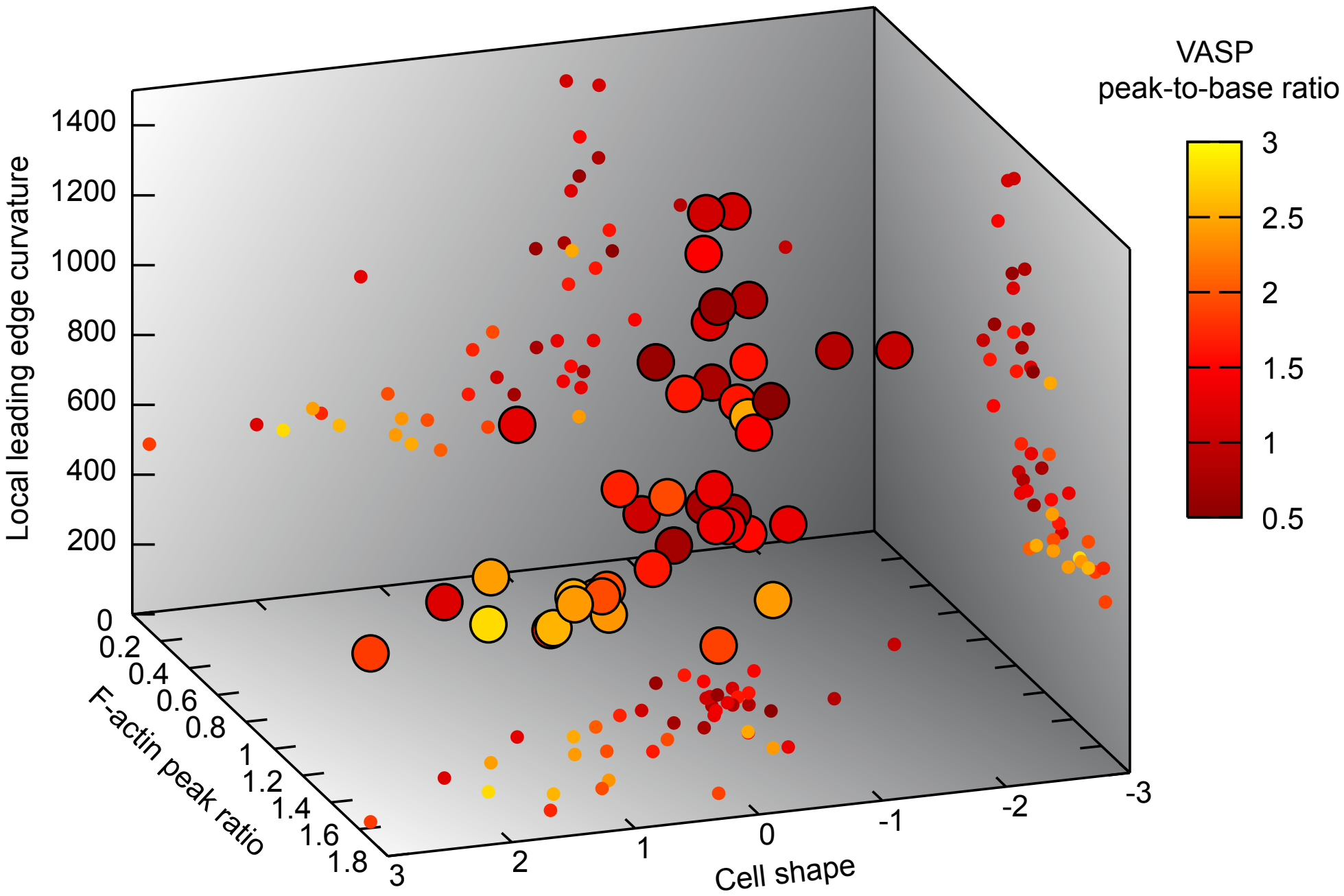
VASP
peak-to-base ratio
3
2.5
2
1.5
1
0.5
Local leading edge curvature
1400
1200
1000
800
600
400
200
0
F-actin peak ratio
0.2
0.4
0.6
0.8
1
1.2
1.4
1.6
1.8
Cell shape
3
2
1
0
-1
-2
-3
