# Supplementary material for: Emergence of Large-Scale Cell Morphology and Movement from Local Actin Filament Growth Dynamics
Source: PLoS Biol. 2007 Aug 28;5(9):e233. doi: 10.1371/journal.pbio.0050233 (PMC1951782; doi:10.1371/journal.pbio.0050233)
Supplement: Supplementary file 4 [file pbio.0050233.sd001.doc]

**Supporting Text: Details of the Mathematical Model**

**Mathematical model: theory**

*1. F-actin density and lateral flow at the leading edge*

We model the densities of the right- (left-) oriented growing barbed ends along the leading edge with functions:

() for ends not associated with VASP and

() for ends associated with VASP.

According to the model assumptions, the following equations govern these densities:

(1)

Here the lateral flow rate *V* is not constant, but rather is proportional to the local actin growth rate. However, in the relevant limit, its exact value does not matter for the approximate solution, and only the order of magnitude is important, so for most calculations we kept *V* constant on the order of the cell speed. Strictly speaking, the spatial variable x here has to be the arc length along the leading edge, but because the edge is not highly curved, we considered x to be the coordinate along the axis normal to the direction of migration. represent the constant rates of capping and VASP association/dissociation, respectively. is the total number of nascent filaments branched out along the leading edge per second. Note that the VASP concentration is in the model implicitly, reflected in parameter quantifying the rate of binding of VASP to the growing barbed end. G-actin concentration is also in the model implicitly (the protrusion speed depends on it), and it appears explicitly below in the model for the shape of the leading edge.

We consider these equations on the leading edge: and we choose the boundary conditions atas follows:

(2)

These conditions are discussed in detail below.

We first use perturbation theory to solve equations (1-2) analytically; then we solve them numerically to verify the analytical solutions. First, to nondimensionalize equations (1-2), we choose characteristic scales as follows. Natural length scale is the length of the cell leading edge, 2*L*. We choose the characteristic time of capping, , as the time scale. Finally, we choose the total number of nascent filaments branched out per unit length of the leading edge over the characteristic time scale, , as the filament density scale. This allows us to rescale the equations introducing nondimensionalized time, distance, and densities: ,,, respectively. (Here, *b* stands for all four densities). We use italic and nonitalic letters to denote nondimensional and dimensional variables, respectively. Substitution of these nondimensional variables into equations (1-2) leads to the nondimensional system:

(3)

(4)

(Note, that the scaling here is slightly different from that used in **Figure 6** in the main text, where we used the half length of the cell leading edge, *L*, as the length scale, so the sides were characterized by ±1, not by ±1/2). The system’s behavior is characterized by a small number of the nondimensional parameter combinations:

(5)

The value of is crucial. The capping rate, , is of the order of 1/s (for relevant discussion see [1]), the lateral flow rate , and half lamellipodial length ; in the biologically relevant regime, barbed ends are capped in seconds, long before they move laterally across the leading edge, so. We first consider the limit in which VASP kinetics is much faster than capping: . In this limit, the two last terms in the right hand sides of equations (3) describe processes on the fast time scale, while, as it is shown below, both advection terms, and the sum of termsare responsible for the slow processes. Thus, VASP association/disassociation equilibrates rapidly, so the quasi-state approximation, , can be used, and the number of VASP-associated barbed ends is linearly proportional to the number of uncapped ends without VASP: . We are interested in the steady state actin distribution, so the first of equations (3) becomes: . Taking into account that , this equation can be rewritten as .

Furthermore, .

Introducing

(6)

we obtain two steady state equations:

(7)

Note that due to the smallness of parameter , equations (7) imply that , so in the steady state, and, meaning that local densities of the left- and right-oriented filaments are almost equal. Below, we confirm these approximate equalities numerically.

The quasi-linear equations (7) can be solved with the standard substitution, that turns differential equations (7) into the algebraic system: . This system has a nontrivial solution if the condition is satisfied, leading to the following equation:

(8)

Below, we demonstrate that , and so. Equation (8) allows two solutions: ,.

Thus, equations (7) have the following solutions: . Due to the symmetry of the problem, , and

(9)

Substituting (9) into (6), using (8) and using the boundary conditions (4), we find the system of three algebraic equations for three unknown variables,:

(10)

or (11)

(12)

From (12), , substituting which into (10), we obtain: . Finally, using (11), we obtain the equation for:

(13)

This equation can be solved graphically (**Figure A**). Note that the left hand side of (13) is convex down and at small has asymptote , while the right hand side is convex up and at small has asymptote , so equation (13) has a nontrivial solution only when (**Figure A**). In this case, the steady actin distribution has a minimum at the center of the leading edge, which is not observed. The dynamics of cell behavior provide a simple explanation as to why this distribution is not observed as a stable state for our measured population of polarized cells. When the leading edge becomes too long, the F-actin distribution is expected from our analysis to become concave (and , transiently) because the diminished number of filaments at the center of the leading edge cannot effectively push forward. As a result, the center of the lamellipodium starts to lag behind and eventually collapses, and the left and right halves of the lamellipodium evolve into two separate lamellipodial protrusions. This phenomenon is observed, though rarely (less than 1% of unperturbed cells), and we will not treat it further.

Note also, that if, then,, and .

**Figure A. Graphical solutions of equations (13, 19).** Left: graphical solution of equation (13). Solid curve is the right hand side of this equation,. Dashed curves are the left hand sides of this equation corresponding to (lower curve) and(upper curve). Right: graphical solution of equation (19). Solid curve is the right hand side of this equation,. Dashed curves are the left hand sides of this equation corresponding to (lower curve) and(upper curve).

When, it turns out that is an imaginary number:

, or

(14)

Then, , and

(15)

(sindisappears due to the symmetry).

Substituting (15) into (6), using (14) and the boundary conditions (4), we find the system of three algebraic equations for three unknown variables, :

(16)

or (17)

(18)

From (18),, substituting which into (16), we obtain: . Finally, using (17), we obtain the equation for:

(19)

This equation can be solved graphically (**Figure A**). Note that the left hand side of (19) is convex up and at small has asymptote , while the right hand side is convex down and at small has asymptote , so equation (19) has a nontrivial solution only when (**Figure A**). In this case, the steady actin distribution has a maximum at the center of the leading edge, in agreement with the observations. The nonlinear algebraic equation (19) can be solved numerically, using, for example, the bisection algorithm. Such solution reveals that when parametervaries from 0 to 0.45, parameter varies from to 1 justifying the assumption that . In this range, the solution is well approximated by the function .

To support the analytical results, we first solved equations (6, 7) numerically using standard methods of numerical analysis described previously[1]. **Figure B** illustrates the numerical solutions and corresponding analytical approximations showing excellent agreement of the analytical and numerical solutions.

Next, we solve numerically the system of equations (3-5). The simplest case corresponds to the following boundary conditions:

, .

**Figure B. Numerical solutions of equations (6, 7).** Steady state solutions (obtained after 200 dimensionless time units) are shown, ; solid/dashed curves correspond to the densities of the right-/left-oriented filaments, respectively. Circles correspond to the analytical solutions characterized by the respective values of the parameters and shown in the figure.

In this case, and when, the following approximate analytical solution holds: .

**Figure C** illustrates the numerical solutions. **Figure C.1, C,2** demonstrates the validity of the analytical approximation and also show that the assumption is not crucial for the validity of the analytical approximation. **Figure C.2** depicts the case in which this assumption holds, while in **Figure C.1**, , but all four steady actin densities are almost indistinguishable between the two figures.

We observed in the barrier experiment with a microneedle stalling the leading edge that VASP associates with the leading edge on the scale of seconds, and so it has to dissociate on the same scale in order to adjust fast to the dynamic actin density. Therefore, it is likely that . We assume that is proportional to VASP concentration, so that VASP reacts with the barbed ends according to the simple mass action law.

In order to investigate the influence of the boundary conditions on the steady actin distributions, we solve numerically the system of equations (3-5) with various parameters . Comparison of **Figure C.1, C.3** shows that if the value of parameter is such that, then the steady state solutions do not have boundary layers, and are well approximated by the cosine-like shapes for all values of *x* (**Figure C.1**). Changing the value of parameter introduces boundary layers in which the steady state actin density rapidly changes (**Figure C.3**). Biologically, these boundary layer effects are not likely to be important. However, the values of parameters regulate the overall shape of the actin distribution. Indeed, as seen from **Figure C.4, C.5**, keeping ensures that the total F-actin density at the boundaries of the leading edge is approximately the same when the value of parametervaries. But when , the total F-actin density at the boundaries of the leading edge increases as grows proportionally to its increase at the center of the leading edge. In the future, we plan to compare the differences in the F-actin profiles changing with time at the leading edge of live cells, as well as those between fixed cells at the same time, to clarify the nature of the boundary conditions.

**Figure C. Numerical solutions of equations (3-5).** Steady state solutions (obtained after 200 dimensionless time units) are shown, . In panels 1-3, solid/dashed lines correspond to the densities of the right-/left-oriented filaments, respectively. Thick/thin curves are the densities of VASP-associated/VASP-free filaments, respectively. Panels 4-5 show the total actin densities at various VASP levels and two different boundary conditions.

In **Figure 6C** in the main text, we plotted the numerical solutions for the total density of uncapped barbed ends along the leading edge, , when the steady state was achieved, demonstrating very good agreement with observations: at high VASP activity (greater values of ) the F-actin density at the sides does not change much (**see Figure 6C,** main text), but increases at the center, and the F-actin profile looks like an inverted parabola (see **Figure 5C, 6C,** main text).

The biological meaning of the boundary conditions is that at the sides of the leading edge the cell, where large cell adhesions are located, there are specific local conditions generating and maintaining constant densities of uncapped barbed ends. The right/left-oriented filaments glide laterally to the right/left, so the corresponding density has to be defined only at the left/right. We assume that these conditions at the sides are uncoupled from the actin dynamics along the leading edge. One further possible assumption is that the “age” of any right-oriented filaments at -*L* is zero, since they immediately glide to the right away from the side. Therefore, if all new filaments are “born” VASP-free, then all right-oriented filaments at -*L* must be VASP-free. This argument and similar reasoning for the left-oriented filaments at the right side lead to the boundary condition . Another possibility is that VASP equilibrates with the actin dynamics at the cell sides according to the same kinetics, , as that at the leading edge. The boundary condition corresponds to this case.

The dimensionless formula and its dimensional form has important biological implications. The stationary F-actin density has an important scaling property that can be understood from the following analysis. First, it shows that the F-actin density depends on the ratio x*/L* of the spatial coordinate to the lamellipodial length, rather than on x and *L* separately, so if the density and spatial coordinate are normalized by the maximal density and the lamellipodial length, respectively, then the F-actin profiles would look the same for all cells, which is observed experimentally (**Figure D**).

Second, as seen from **Figure C.4, C.5**, an increase of parameterincreases the maximal total F-actin density at the center and curvature of the F-actin distribution at the leading edge (for both explored boundary conditions; the effect of increasing the curvature is more pronounced when ). So, assuming that is an increasing function of VASP concentration, the maximal F-actin density at the front center of the cell is proportional to the levels of VASP.

**Figure D. Observed F-actin densities along the leading edge of keratocytes.** The F-actin distributions along the leading edge of keratocytes with high VASP peak-to-base ratios and different cell widths stretch proportionally. Cells widths are specified in the legend.

According to the model used in this study, which is similar to the “global” model discussed previously[1], a global factor (like G-actin density, for example) determines the total number of nascent filaments appearing per second per whole leading edge. In this case, “each filament has an equal probability of branching per unit length independent of the filament’s location”[1]. The alternative “local” model proposes a constant local branching rate per unit length of the cell boundary but lacks a scaling property: when the leading edge length increases (larger cell widths), F-actin profiles do not scale and become flat at the center with constant slopes at the sides [1]. Our observations contradict this prediction of the “local” model (**Figure D**). Also note that our model implicitly assumes that branching occurs either at the tips of the growing filaments, or at the sides of filaments *in close proximity to the growing tip* (this mechanism has been deemed to be the most plausible scenario [2]). If branching instead took place *anywhere* on the sides of filaments, there would still be one ‘daughter’ filament per ‘mother’ filament on average [2]; therefore, no change in the steady state model equations employed here would be required. In non-steady state situations, the model equations would have to be changed to keep track of both filament lengths and capped/non-capped filaments. This would complicate the analysis considerably; however, our results would not change qualitatively, as they concern mostly steady state situations examined experimentally.

2. *Density-protrusion velocity relation at the leading edge*

Growing barbed ends elongate with the rate:

(20)

limited by two factors: membrane resistance per filament, *f*, and local G-actin concentration, *g* [3]. Here, is the thermal energy, is the distance of the order of actin monomer size, and the characteristic force generated by one growing filament is represented by the ratio:

.

The force per filament can be expressed in the form:

(21)

where is the uncapped barbed end density per micron of the leading edge defined above, while *F_r_* is the membrane resistance force per micron of the leading edge. When we introduced the notation:

, then the normal local protrusion rate was:

. (22)

In order to estimate the local G-actin concentration,, at the leading edge, let us consider first how the G-actin concentration changes in the direction normal to the leading edge. Let us introduce , where *y* is the coordinate in the direction normal to the leading edge (so that ) and assume that the G-actin concentration changes slowly, on the scale of tens of microns, along the leading edge, and rapidly, on the scale of microns, in the direction perpendicular to the leading edge [3]. This assumption is justified because the F-actin density is changing slowly, on the scale of tens of microns, along the leading edge, but the structure and dynamics of the F-actin network is graded on the scale of microns in the direction perpendicular to the leading edge [3].

The diffusive flux of G-actin at the leading edge, (*D_m_* is the actin monomer diffusion coefficient), is determined by the quantity of actin monomers assembling per second onto growing barbed ends at the leading edge, which is proportional to the local rate of protrusion and barbed end density: [3] ( is the proportionality coefficient determined by the lamellipodial geometry). By solving this equation, we estimated the gradient of the G-actin concentration at the leading edge: . (). Most F-actin disassembles within *l* = a few microns from the lamellipodial leading edge [2], so: where and is the G-actin concentration a few microns behind the leading edge.

To estimate , note that it is roughly proportional to the local F-actin concentration at the leading edge, because almost all actin filaments disassemble into monomers, so , where is the proportionality coefficient. Thus, (23)

The important conclusion of this formula is that the local G-actin concentration at the leading edge is proportional to the local F-actin density, because both sink and source of G-actin are local assembling/disassembling actin filaments, respectively. Substituting (23) into (22), we obtain, after elementary algebra, the density-velocity relation:

. (24)

Here is the order of magnitude of the protrusion rate, , and is the geometric factor estimated previously to be of the order of 1-10 per micron [3]. We choose . The density-velocity relation (24) shown in **Figure E** has peculiar biphasic behavior: when the number of the barbed ends is small, 50/*µm* or less, then the membrane resistance limits the protrusion, and the protrusion rate is the rapidly changing increasing function of the number of the barbed ends. On the other hand, when the number of the barbed ends is great, 50/*µm* or more, then the filaments easily overcome membrane resistance, and the G-actin density starts to limit the protrusion, and the protrusion rate saturates and becomes insensitive to the number of the barbed ends.

**Figure E. Computed protrusion rate as a function of barbed end density.** At high barbed end density (“coherent” regime), the protrusion rate is not sensitive to the number of barbed ends, so fluctuations in F-actin density do not affect protrusion. On the other hand, at low barbed end density (“decoherent” regime) protrusion rate is limited and sensitive to the number of barbed ends at the leading edge.

*3. Stability of the leading edge*

We describe the leading edge profile with the function: . Mathematically, local fluctuations of the leading edge profile around the smooth average shape can be described with the equation:

(25)

where is the steady average barbed ends density corresponding to the steady average leading edge profile, which changes smoothly on the long spatial scale, but locally can be considered constant. is the local barbed ends density fluctuating on the short spatial scale, and the first term describes appearance of local protruding “lobes” at the leading edge due to local increase in the barbed ends number. Parameter is the derivative of the protrusion rate (24) with respect to the barbed ends density; it is great at small (10 - 50/*µm*) values of and small at high (> 10 - 50/*µm*) values of . The second term describes the correction of the protrusion rate due to the local membrane curvature, . If a protrusive lobe develops, , and there is a restoring force trying to flatten the membrane, while if there is a local indentation in the leading edge (part of the leading edge lags behind), , then membrane tension at the sides restores flatness helping protrusion. Because membrane resistance is proportional to the Gaussian curvature at the leading edge [4], the main component of which is the high curvature in the dorsal-ventral direction, the second term in equation (25) responsible for the lateral bending of the leading edge is very small, and so the coefficient *r* is very small.

In order to quantify local oscillations of the actin density at the leading edge, the rate of lateral flow of barbed ends has to be analyzed in detail. Let be cell speed. Then, if the leading edge is exactly perpendicular to the direction of the cell’s crawling (mathematically, ), then the rate of the lateral flow for the right-oriented filaments is [1]. However, if the leading edge is tilted so that the right side of the edge is advancing more than the left side, , the right-oriented filaments flow to the right faster, at the rate , than the left-oriented filaments flowing to the left slower, at the rate . Let us introduce the notations: . Then, equations (3) for the growing barbed ends’ densities have additional drift terms at the right hand sides when the leading edge is slightly tilted:

(26)

Same as above, . For simplicity, here we consider the limiting case when VASP kinetics are fast and densities are in quasi-steady-state equilibrium with . Also for simplicity, we do not explicitly account for in the full actin density —the model conclusions do not change qualitatively because of this. Introducing the full actin density, , and the difference between right- and left-oriented densities, , and adding and subtracting equations (26), we find:

(27)

Let us regroup the second equation from system (27) as follows: . We know from the analysis above that , while . Therefore, the first equation from system (27) describes a slow dynamics of the full actin density, while the second equation accounts for a rapid relaxation of the variable *s* to the quasi-steady state that can be found as follows: ,, so

(28)

Substitution of (28) into the first equation from system (27) results in the equation:

, (29)

so the local barbed ends dynamics can be described by the equation:

(30)

Here the first two terms are responsible for the branching/capping processes and effective barbed ends diffusion due to the lateral flow, respectively. The third term is responsible for the effective net lateral flow of F-actin due to the local bending of the leading edge. This term, in fact, describes effective focusing of the F-actin density into protruding lobes at the leading edge: at the left side of the lobe (where ) the barbed ends slide to the right, toward the lobe’s center, and at the right side of the lobe (where ) the barbed ends slide to the left, again toward the lobe’s center. This process potentially can destabilize the smooth leading edge making it rough, because greater number of the barbed ends focusing into the nascent lobe would push the lobe forward faster creating the positive feedback.

To make this argument precise, we investigate the stability of the solutions of equations (25, 30). The flat leading edge with constant local barbed end density, is the steady state of this system. We introduce local deviations of the barbed ends density from the average, , and rewrite (25, 30) as:

(31)

(Note that).

We look for sinusoidal perturbations of the F-actin density and leading edge shape in the form:

(32)

where and are the amplitudes of heterogeneities in shape and density, respectively, is the rate of growth of the perturbation, and 1/*q* is the wavelength of the perturbation (biologically, the size of the protruding lobe).

Substituting (32) into (31), we obtain the system of linear algebraic equations for the perturbation amplitudes:

We are interested in the case when the size of the protruding lobe is much smaller than the length of the leading edge, so, and the last system can be simplified:

This system of equations has nontrivial solutions under the condition:

that provides the following dispersal relation:

(33)

One of the perturbation amplitudes (corresponding to the minus sign) always decreases with time, while another (corresponding to the plus sign) can grow if is positive. We are interested in the following characteristic parameter values: (corresponding to the lobe size ~1µm. Large values of *q* mean short-scale heterogeneities at the leading edge; relevant spatial scale is microns —much greater than the distance between filaments and much smaller than the leading edge length). We plot the upper brunch of given by (33) as a function of for two plausible values of parameter *r* in **Figure F**.

In the regime of a low F-actin density (50 or less barbed ends per micron of the edge), the slope of the protrusion rate – F-actin density relation is steep (**Figure E**), and parameter is large. Then, perturbations will grow () while the leading edge looses stability and becomes rough. On the other hand, in the regime of a high F-actin density (50 or more barbed ends per micron of the edge), the slope of the protrusion rate – density relation is very shallow (**Figure E**), and parameter is very small. Then, the growth rated of perturbations growth rate negative () and the smooth leading edge remains stable.

**Figure F. Growth rate of the leading edge perturbation (33).** The growth rate given by (33) is plotted as the function of parameter when for two plausible values of parameter *r*.

*4. Global shape of the leading edge*

Using formulae (15) for the barbed ends distribution along the leading edge and (24) for the normal protrusion rate as function of the barbed ends distribution, we can compute the shape of the leading edge from the Graded Radial Extension model[1,5], according to which , where is the orientation of the normal to the leading edge at position x. Expressing in terms of the second derivative of the function describing the leading edge shape allows us to derive the differential equation describing the profile of the leading edge:

, (34)

We solved equation (34) numerically using formulae (15, 24) with the boundary condition: . In the calculations, we used the following parameters and functions:

for decoherent, rough cells (corresponds to ~15 *µm*, barbed ends density at the center is 50/*µm*, and the F-actin density is essentially flat), and

for coherent, smooth cells ( corresponds to the lamellipodial width being ~30% greater than that of decoherent cells; barbed ends density at the center is , twice that of decoherent cells, and the F-actin density is peaked at the center). The resulting computed shapes are shown in **Figure 6D** (main text) and look very similar to the observed ones. The F-actin density peaks at the center in coherent cells, and at this high actin density, the protrusion rate, insensitive to the barbed ends density (**Figure E**), decreases very slowly toward the sides, so the leading edge remains smooth and flat and extends far from side to side (see **Figure 6D,** main text). At the sides, where the F-actin density decreases significantly, membrane resistance starts to limit protrusion, and the rapidly decreasing protrusion rate leads to high curvature at the sides of the leading edge.

Note that though smooth cells have wider leading edges, the *arc* length of the edge of rough cells is actually greater due to the large total perimeter of their irregular protruding lobes (**Figure G**). This factor decreases the number of the barbed ends per micron in decoherent cells and further slows these cells down and deregulates their front. The overall shape of the leading edge in decoherent cells remains parabolic (see **Figure 6D,** main text), though it becomes narrower, with sharper transition from the flat center to the curved sides, because actin protrusion decreases faster from the center to the sides effectively rapidly increasing the overall leading edge curvature apparent as the rounder “D” keratocyte shape.

Note, that the dimensional formula for the total F-actin density at the leading edge has the form: ,

where , and dimensionless parameter. The actual F-actin distribution depends on the total branching rate, , leading edge length, , capping rate, , the boundary density, , and VASP association rate, , proportional to the concentration of VASP. Regulation of the cell shape, in particular of the leading edge length, and other parameters, is beyond the framework of this paper; however, the following argument seems reasonable. Let us assume that parametersanddo not change when the VASP concentration at the leading edge increases. The leading edge length,, of coherent cells is ~20% greater than that of decoherent cells (see **Figure 7B,** main text), but the arc length of decoherent cells is ~20% greater than that of coherent cells (**Figure G**). Assuming that the total number of branching events per second over the whole leading edge does not changes when the VASP concentration at the leading edge increases, parametercorresponding to the coherent cell is effectively ~20% greater than that corresponding to decoherent cells, because of the geometric effect of the projection of the branching event density from the rough edge onto the x-axis parallel to the overall leading edge. Then, in both coherent and decoherent cells, parameter is roughly the same ( are the same, while both and are ~20% greater in the coherent cell). Therefore, parameter is roughly the same in both coherent and decoherent cells, and, due to the factor , an increase in the concentration of VASP increases the steepness of the F-actin distribution at the leading edge.

**Figure G. Observed leading edge arc length of the leading edge as a function of leading edge curvature.** The leading edge arc lengths of keratocytes correlate with their local leading edge curvature. At high local leading edge curvature (“rough”), the arc length is ~20% greater than that at low local leading edge curvature (“smooth”). Note that “rough” cells are shorter from side to side, so their greater leading edge arc length is due to the geometric effect of the increased total perimeter due to the multiple small ‘lobes’ at the rough leading edge.

# Mathematical model: limitations and future work

# The model described above gives but the first, semi-quantitative explanation of the observed phenomena. To achieve a deeper quantitative understanding, the following modeling assumptions will have to be made more realistic, and the following extensions will have to be made: (i) Continuous angular distribution of growing actin filaments has to be introduced and simulated. (ii) Explicit 2D model for G-actin turnover has to be coupled to the F-actin growth model, and both have to be simulated simultaneously. (iii) Systematic parameter search (and search for adequate force-velocity relations and reaction kinetics) accompanied by comparisons of theoretical results to quantitative data will make the model comprehensive. These changes will be best handled in the framework of Monte-Carlo-type, stochastic, agent-based computational models, rather than one based on differential equations. The latter would become hopelessly complicated even with an additional angular variable and miss potentially important stochastic effects to boot. The problem of F-actin organization at the leading edge and edge shape is coupled to the more general problem of shape regulation of the whole motile cell, and at some point this coupling will have to be made computationally. Finally, future computations have to go hand-in-hand with quantitative experiments, without which modeling progress will be mired hopelessly in too many theoretical possibilities.

References

1. Grimm HP, Verkhovsky AB, Mogilner A, Meister JJ (2003) Analysis of actin dynamics at the leading edge of crawling cells: implications for the shape of keratocyte lamellipodia. Eur Biophys J 32: 563-577.

2. Carlsson AE, Wear MA, Cooper JA (2004) End versus side branching by Arp2/3 complex. Biophys J 86: 1074-1081.

3. Mogilner A, Edelstein-Keshet L (2002) Regulation of actin dynamics in rapidly moving cells: a quantitative analysis. Biophys J 83: 1237-1258.

4. Gov NS, Gopinathan A (2006) Dynamics of membranes driven by actin polymerization. Biophys J 90: 454-469.

5. Lee J, Ishihara A, Theriot JA, Jacobson K (1993) Principles of locomotion for simple-shaped cells. Nature 362: 167-171.
